# Drug-Induced Hyponatremia: Insights into Pharmacological Mechanisms and Clinical Practice Management

**DOI:** 10.3390/jcm14186584

**Published:** 2025-09-18

**Authors:** Miguel Capinha, Marta Lavrador, Joana Liberato, Adriana Pinheiro, Ana Aveiro, Isabel Vitória Figueiredo, Margarida Castel-Branco

**Affiliations:** 1 Pharmacology and Pharmaceutical Care Laboratory, Faculty of Pharmacy, University of Coimbra, Polo das Ciências da Saúde, Azinhaga de Santa Comba, 3000-548 Coimbra, Portugal; mcapinhad@gmail.com (M.C.); isabel.vitoria@ff.uc.pt (I.V.F.); mmcb@ci.uc.pt (M.C.-B.); 2 Coimbra Institute for Clinical and Biomedical Research (iCBR), Polo das Ciências da Saúde, Azinhaga de Santa Comba, 3000-548 Coimbra, Portugal; 3 USF Serra da Lousã, Unidade Local de Saúde de Coimbra, 3200-420 Lousã, Portugal; mjliberato@ulscoimbra.min-saude.pt (J.L.); ampinheiro1@ulscoimbra.min-saude.pt (A.P.); 4 USF As Gândras, Unidade Local de Saúde de Coimbra, 3060-318 Cantanhede, Portugal; 42263@ulscoimbra.min-saude.pt

**Keywords:** hyponatremia, drug-related side effects and adverse reactions, water–electrolyte balance, pharmacological mechanisms, clinical management

## Abstract

**Background:** Hyponatremia (serum sodium concentration < 135 mmol/L) represents the most common electrolyte disturbance in clinical practice, particularly among high-risk populations such as older adults. Its severity ranges from moderately severe to life-threatening symptoms, contributing to increased mortality. Its etiology is widely heterogeneous and leads to different classifications according to volume status such as hypovolemic, euvolemic and hypervolemic hyponatremia. Drug-induced hyponatremia presents itself as one of the most prevalent but frequently overlooked causes, since many confounding factors like associated comorbidities and polypharmacy complicate the identification of specific medicines as the main offenders. **Objectives**: This narrative review was performed to provide a comprehensive analysis on drug-induced hyponatremia, focusing not only on the underlying pharmacological mechanisms, but also on management strategies in clinical practice. **Methods**: A narrative literature review was conducted using PubMed, Science Direct and Google Scholar. **Results**: This narrative review focused not only on the most common drug classes to induce hyponatremia through different mechanisms, including diuretics, antidepressants, anticonvulsants, and antipsychotics, but also on other pharmacological classes, that, although to a lesser extent, might also be associated with decreasing serum sodium levels (antineoplastic and immunomodulating agents, drugs acting on digestive and locomotor systems, anti-infective drugs, endocrine diseases drugs, among others). It also explores recommendations on the management of drug-induced hyponatremia and it emphasizes the role of healthcare providers in addressing this electrolyte disorder. **Conclusions**: As drug-induced hyponatremia poses significant challenges in clinical practice, understanding its mechanisms, coupled with effective management strategies, can enhance patient safety.

## 1. Introduction

Having multiple physiological roles within the human body, sodium is essential, not only for cellular signaling and for generating and conducting action potentials in muscles and nerves [1], but also for maintaining electrolyte and water balance [2].

Given that energy-dependent transporters, such as the Na^+^/K^+^ pump, predominantly confine sodium to the extracellular compartment, the serum sodium concentration is intricately linked to the regulation of extracellular water. This regulation hinges on a delicate balance between intake (modulated by the thirst mechanism), antidiuretic hormone (ADH), which promotes the expression of water transport proteins in the late distal tubule and collecting duct, leading to increased water reabsorption [3], and output, primarily governed by renal processes [2].

Serum sodium concentration remains tightly regulated between 135 and 145 mmol/L, exhibiting minimal variations [4]. However, any deviation from the homeostatic balance of water can precipitate pathophysiological processes [5,6]. Consequently, hyponatremia results from the excess of total body water when compared to the total amount of sodium [3,5].

Hyponatremia, which has a prevalence of approximately 8% in the community [7,8], is the most common electrolyte disturbance in clinical practice [9,10]. Among hospitalized patients, 40% to 70% of cases are iatrogenic in origin, with the variation reflecting differences in study populations and hyponatremia classification [10,11]. Although hyponatremia is frequently undercounted [4], its noteworthy impact is evident, accounting for around 20% to 35% of hospital admissions, being particularly pronounced among individuals of advanced age, who often present with multiple comorbidities, are on several medications, and have impaired mechanisms for water excretion and intake [5,7].

Drug-induced hyponatremia is often underdiagnosed due to its multifactorial nature [12]. Early identification and intervention are critical to preventing severe complications, including an increased mortality risk.

Previously published studies have addressed drug-induced hyponatremia, with several focusing specifically on its clinical diagnosis and management, and others exploring the pharmacological and pathophysiological mechanisms of the drugs implicated [13,14,15,16,17,18,19]. This narrative review consolidates findings from these distinct sources, drawing from multiple studies centered on drug-induced hyponatremia, diagnostic and therapeutic approaches, and mechanistic insights to provide a comprehensive clinical resource. Its main contribution is the integration of these perspectives, with particular emphasis on elucidating mechanisms and guiding management strategies.

## 2. Methods

A comprehensive, non-systematic literature search was performed using PubMed, ScienceDirect, and Google Scholar. The primary search covered the period 2015–2025 to capture the most recent and clinically relevant evidence. Search terms included (“Hyponatremia” [tiab]) OR (“Drug-induced hyponatremia” [tiab]) OR (hyponatremia [tiab] AND drug-induced [tiab]) OR (hyponatremia [tiab] AND management [tiab]). Particular attention was given to clinically relevant systematic reviews, meta-analysis, practice guidelines, consensus statements, original research articles, case reports, and editorial letters.

Earlier landmark publications were also included when identified through supplementary targeted searches or by screening the reference lists of relevant studies, to ensure completeness. This approach enabled a more in-depth analysis of the literature on specific drug classes of interest.

Non-English articles were excluded. No formal review protocol or standardized critical appraisal tool was applied, consistent with the narrative and qualitative nature of this synthesis. In total, 183 publications were included.

## 3. Hyponatremia: Definition and Classifications

Hyponatremia is the most common disorder of body fluid and electrolyte balance in clinical practice and is defined as a serum sodium concentration < 135 mmol/L [3]. Given the heterogeneous nature of hyponatremia [6], classification can be based on various parameters. The European Clinical Practice Guideline on the Diagnosis and Treatment of Hyponatremia outlines these classification criteria, which are summarized in Table 1 [3].

As shown in Table 1, hyponatremia can also be classified as hypotonic or non-hypotonic based on effective osmolality. This differentiation holds significant clinical importance as the management strategies for each disorder differ [6]. Since hypotonic hyponatremia is diagnosed only after non-hypotonic causes have been excluded, understanding the factors that determine each type is crucial for optimal management [20].

Non-hypotonic hyponatremia occurs due to the presence of active osmoles other than sodium, such as glucose or mannitol, leading to a non-hyposmolar state. This results in the dilution of serum sodium concentration, but without the risk of brain edema and its associated symptoms [3].

In hypotonic hyponatremia, low serum sodium concentration is accompanied by low serum osmolality, which explains characteristic symptomatology [21,22]. It manifests as an excess of free water, which can result from increased water intake or decreased renal water excretion [5].

Differentiation among the various types of hypotonic hyponatremia (hypervolemic, hypovolemic or euvolemic based on volume status) can typically be achieved through a combination of patient history, physical examination, and comprehensive laboratory results [20]. While hypervolemic hyponatremia often manifests with obvious signs such as ascites or edema, distinguishing between hypovolemic and euvolemic states presents a challenge in clinical practice. This complexity arises due to the wide spectrum of underlying diseases associated with these three volemic states [6]. Euvolemic hyponatremia is very common in hospitalized patients and is mostly attributed to the Syndrome of Inappropriate Antidiuretic Hormone Secretion (SIADH). This syndrome is predominantly caused by malignancy, medications, and central nervous system disorders [20,23]. However, diagnosing SIADH requires the exclusion of other conditions that may alter ADH levels.

To enhance the understanding of the clinical presentation and underlying etiologies, Table 2 provides a comprehensive summary of the information for each type of hypotonic hyponatremia, as outlined in the European Guidelines and the review on the diagnosis and management of hyponatremia by Adrogué et al. [3,20].

## 4. Drug-Induced Hyponatremia

Older adults are particularly susceptible to electrolyte disturbances due to factors like age-related impairment in water excretion, multiple comorbidities, and polypharmacy [5,7,24]. Regarding this, it is crucial to assess patients’ medications during the medical history review, as drug usage remains one of the most prevalent yet rarely identified causes in hospital settings [15,25].

Physicians should be aware of drugs that can cause hyponatremia, as pharmacological causes are often overlooked when prescribing [7,12]. It is also important to identify drug combinations that can markedly increase the risk of electrolyte disorders, sometimes up to ten-fold [12].

Most drug-induced hyponatremia cases occur within the first weeks of treatment. When patients present with low serum sodium concentrations after receiving a drug for a prolonged period, other underlying factors should be considered before discontinuing the medication, as well as performing a benefit–risk analysis [7].

A prospective pharmacovigilance program found that severe hyponatremia is often due to medication. Evaluation of this electrolyte disorder as an adverse drug reaction revealed that 70% of the cases represent hypovolemic hyponatremia (based on isotonic saline response), most commonly associated with diuretic use and the remaining 30% represent SIADH. The leading causes of drug-induced severe or very severe hyponatremia were thiazides and thiazide-like drugs, such as indapamide and chlortalidone (22.6% of cases in fixed-dose combination with potassium-sparing diuretics or other antihypertensives) and loop diuretics (9.2%), causing hypovolemic hyponatremia. SIADH was the second most frequent diagnosis, caused primarily by antidepressants (9.3%), anticonvulsants, and antipsychotics (together representing a total of 8.7%) [24].

### 4.1. Main Mechanistic Pathways

From a pharmacological perspective, a variety of mechanisms can contribute to drug-induced hyponatremia by promoting sodium depletion or water retention through interactions with multiple physiological systems.

In the central nervous system, certain drugs may induce sodium loss through mechanisms such as hyperhidrosis (which enhances sodium loss and worsens hyponatremia) [15,26,27]. However, the predominant mechanism by which CNS-acting drugs induce hyponatremia is water retention, most often through SIADH. Some medications stimulate hypothalamic thirst mechanisms or exert anticholinergic effects, thereby increasing free water intake and retention, ultimately lowering serum sodium levels [28,29].

The most significant and well-documented central mechanism by which drugs induce hyponatremia is SIADH, which can be triggered through different pathways. As a syndrome of inappropriate antidiuresis (SIAD), SIADH results from the inappropriate secretion of ADH due to the inability to suppress it via negative feedback mechanisms. In this context, ADH secretion is not inhibited despite a decrease in serum osmolality or changes in circulating volume regulation [20,23]. Some drugs exert direct/toxic effects on the hypothalamus and neurohypophysis, stimulating ADH production and release [30], while others lower the osmotic threshold for ADH secretion [31].

Neurotransmitters such as serotonin, norepinephrine, dopamine, gamma-aminobutyric acid (GABA), and glutamate are believed to modulate ADH release via their respective receptors located on vasopressinergic neurons in the hypothalamus and in neurohypophysis. This receptor-mediated activation leads to stimulation of ADH secretion and reduces the osmotic threshold required for its release [29,32,33]. Notably, this enhances renal responsiveness to ADH, worsening water retention [31]. Drug-induced volume depletion activates baroreceptor-mediated compensatory mechanisms, leading to non-osmotic ADH secretion, thereby exacerbating water retention [34]. Additionally, since inhibition of angiotensin-converting enzyme is not centrally mediated, angiotensin I can be converted to angiotensin II in the brain, stimulating thirst mechanisms and the release of vasopressin [15,34,35].

Drugs affecting the renal system primarily act by blocking, inhibiting or even damaging transporters and channels responsible for sodium retention (such as Na/Cl co-transporter (NCC), the Na-K-2Cl co-transporter (NKCC2), the epithelial sodium channel (ENaC) and the sodium-potassium pump (Na^+^/K^+^-ATPase)) [14,15,19,36,37,38,39]. Their inhibition reduces sodium reabsorption, leading to its renal loss. This electrolyte depletion might be accompanied by volume loss, triggering non-osmotic ADH secretion, which further exacerbates hyponatremia [14,15,40]. Drugs that downregulate mineralocorticoid receptors or inhibit aldosterone binding also contribute by impairing aldosterone-mediated sodium reabsorption.

Moreover, renal pathologies that impair kidney function can significantly reduce serum sodium levels. In nephrotic syndrome, defective renal tubules impair sodium excretion [3], while hypoalbuminemia lowers oncotic pressure, stimulating vasopressin release and thereby promoting water retention [41]. Nephrogenic syndrome of inappropriate antidiuresis (NSIAD) is a rare genetic disorder that exhibits similar laboratory findings to SIADH, but occurs without any elevation in ADH secretion [14]. Certain drugs can induce an NSIAD-like state by interacting with V2 receptors (V2R) in the kidneys or by increasing their mRNA expression, which leads to aquaporin 2 (AQP2) upregulation or trafficking into the apical membrane of renal collecting ducts, consequently increasing free water reabsorption. This effect happens particularly in individuals with V2R polymorphisms [14,42,43,44]. Another possible drug-induced mechanism for AQP2 regulation in the apical membrane, independent of ADH, is through the prostaglandin E2 (PGE2) pathway. Although evidence remains inconclusive, PGE2 plays a crucial role in aquaporin-mediated water retention. Drugs that affect PGE2 can alter AQP2 translocation to the apical membrane [14,45,46]. In ADH absence, PGE2 interaction with EP2/EP4 receptors promotes AQP2 translocation, increasing water reabsorption. Conversely, in the presence of ADH, interaction with EP1/EP3 receptors promotes aquaporin retrieval, reducing water reabsorption [45,47].

Additionally, any drug that interferes with the renin–angiotensin–aldosterone system (RAAS) has the potential to increase the risk of hyponatremia. Hypoaldosteronism, either by reducing aldosterone secretion or by impairing its renal tubular actions (aldosterone resistance), is a relevant cause of drug-induced hyponatremia. This condition results in impaired sodium reabsorption and reduced potassium excretion in the distal nephron, frequently leading to a hypovolemic state. Certain drugs may impair aldosterone synthesis or action (potassium-sparing diuretics, angiotensin II receptor blockers, angiotensin-converting enzyme inhibitors, heparin, trimethoprim, and mineralocorticoid receptor blockers), thereby contributing to hyponatremia in susceptible patients. Recognizing hypoaldosteronism as a distinct mechanism is essential for differentiating it from euvolemic states such as SIADH, since the therapeutic implications differ substantially [34,48,49].

Hormonal regulation plays a critical role in sodium balance. Drugs that affect adrenocorticotropic hormone (ACTH) release or synthesis can cause secondary adrenal insufficiency (SAI) by reducing cortisol production. Since cortisol inhibits vasopressin release via negative feedback, its deficiency induces a SIADH-like effect, causing uncontrolled ADH release [3,50]. Certain drugs might also trigger primary adrenal insufficiency (PAI), a condition leading to glucocorticoid and mineralocorticoid depletion. In addition to the unchecked secretion of ADH due to cortisol deficiency [51], reduced synthesis of mineralocorticoids (aldosterone) impairs sodium reabsorption, causing renal salt wasting, hypovolemia, and further ADH secretion, exacerbating water retention [52,53]. Hypothyroidism, often secondary to hypophysitis, disrupts thyroid hormone regulation, reducing cardiac output. This leads to decreased renal blood flow [54] and reduced glomerular filtration rate (GFR), thereby diminishing water excretion. In chronic conditions, baroreceptor-mediated compensatory mechanisms increase ADH secretion to offset reduced cardiac output. Moreover, elevated urinary sodium concentrations could potentially result in a misdiagnosis of SIADH [3,23,55].

Other mechanisms and associated factors might also lead to hyponatremia. Due to their specificity, they will be discussed within each drug class section to provide a clearer understanding of their individual impact.

Table 3 provides a summary of the main mechanistic pathways and the corresponding drugs or drug classes involved.

The subsequent sections will provide a detailed examination of the different pharmacological classes, the mechanisms through which they induce hyponatremia, and the drug-related management strategies and clinical recommendations reported in the current literature.

### 4.2. Cardiovascular System Drugs

#### 4.2.1. Thiazides and Thiazide-like Agents

Although primarily indicated for hypertension, this class is also used in conditions associated with hyponatremia, such as heart failure or nephrotic syndrome [56]. Due to their mechanism of action, the most common side effects of this drug class are electrolyte disturbances. Hyponatremia typically develops within weeks of initiation, when renal compensatory mechanisms fail [37,56,57]. Thiazides and thiazide-like agents are recognized as the leading cause of severe and very severe drug-induced hyponatremia [24].

Consistent with this burden, a recent new-user cohort reported a two-year cumulative incidence of hyponatremia of 3.83% for bendroflumethiazide (versus a calcium-channel blocker) and 3.51% for hydrochlorothiazide combined with a renin–angiotensin system inhibitor (versus a renin–angiotensin system inhibitor alone). The absolute risk differences were 1.35% (95% CI 1.04–1.66%) and 1.38% (95% CI 1.01–1.75%), respectively, indicating a higher incidence of hyponatremia with thiazide therapy compared with these specific antihypertensive comparators. Risk was highest in the first month after initiation [58]. These findings are consistent with a population-based case–control study of 11,213 patients hospitalized with a primary diagnosis of hyponatremia that found that thiazide diuretics were implicated in more than one in four of these hospitalizations [59]. Clinically, thiazide-induced hyponatremia typically develops shortly after initiating the drug and appears to be dose-dependent [60]. In fact, the referred study found that the risk of hyponatremia was nearly 50-fold increased during the first week of treatment, gradually declining thereafter. Patients with ongoing therapy remained at more than a 3-fold increased risk [13,59].

Mechanistically, thiazide diuretics are more likely than other diuretics to cause hyponatremia because they act primarily on the distal convoluted tubule, a key site for urinary dilution in the kidney [61].

Despite the potential for thiazides to cause hypovolemic hyponatremia due to sodium loss accompanied by water excretion, most patients present clinically as euvolemic [14,47,62].

Primarily, renal mechanisms underlying thiazide-induced hyponatremia are attributed to their action on renal tubules. By inhibiting the NCC and, consequently, sodium and chloride reabsorption, they impair the kidneys’ ability to dilute urine, leading to increased sodium excretion and increased free water retention [14,45,63]. Independent of NCC inhibition, thiazides may also increase water permeability in the collecting ducts through ADH-independent pathways [14,47]. Specifically, thiazides are believed to upregulate AQP2 in the collecting duct, without the involvement of vasopressin, either directly or through the prostaglandin E2 pathway, consistent with drug-induced NSIAD [14,45]. It is also suggested that some patients with thiazide-induced hyponatremia may carry a variant allele of the prostaglandin transporter (PGT) gene, resulting in a decreased ability to transport prostaglandin E2 (PGE2) across the apical cell membrane [14,45].

As a consequence of their pharmacological action, thiazides can cause some degree of volume depletion. However, this is not considered a primary pathophysiological mechanism of hyponatremia, as most studies have not demonstrated elevated ADH in patients with thiazide-induced hyponatremia [14,47].

Secondarily, extrarenal mechanisms may also contribute via reduced solute intake and increased water consumption [14], further impairing free water excretion. Additionally, thiazide therapy often leads to hypokalemia, which can worsen hyponatremia due to transcellular cation exchange [14]. Concurrent administration of a potassium-sparing diuretic like amiloride or spironolactone is sometimes employed; however, it may worsen hyponatremia by prioritizing potassium conservation over sodium [40].

#### 4.2.2. Loop Diuretics

Monotherapy with loop diuretics, such as furosemide or torsemide, is much less likely to induce hyponatremia than with thiazides, since their main effect is in the ascending limb of the loop of Henle, which is a less important diluting site in comparison with the distal tubule [40,61]. In patients with acute heart failure, the risk of hospital-acquired hyponatremia was significantly higher among thiazide users compared with low-dose (OR 2.67; 95% CI 1.13–6.34) and high-dose loop diuretics (OR 2.31; 95% CI 1.50–5.13) [64].

However, cases of hyponatremia have been documented, and it generally presents as hypovolemic. This occurs through their action in the thick ascending limb of the loop of Henle, where approximately 25% of sodium reabsorption takes place [40]. By reversibly inhibiting the NKCC2, these drugs prevent sodium, potassium, and chloride reabsorption. This reduces medullary interstitial osmolality required for water reabsorption and ultimately increases free-water clearance. Paradoxically, this property can also be leveraged as a therapeutic strategy in the management of euvolemic and hypervolemic hyponatremia, as loop diuretics promote hypotonic urine loss. However, if the diuretic-induced natriuresis leads to excessive volume depletion, it may trigger non-osmotic ADH secretion, further worsening hyponatremia in addition to the solute loss [14,15,40].

#### 4.2.3. Potassium-Sparing Diuretics

Potassium-sparing diuretics, such as amiloride (which blocks ENaC) and spironolactone (an aldosterone antagonist), also inhibit sodium reabsorption in the collecting duct. When combined with thiazides, they lead to natriuresis and an increased risk of hyponatremia [14,15,65].

#### 4.2.4. Calcium-Channel Blockers (CCBs)

While diuretics are well-known for inducing hyponatremia, evidence regarding other anti-hypertensive classes remains insufficient [34].

Calcium-channel blockers have peripheral vasodilation properties, acting directly on vascular smooth muscle, with dihydropyridines being more selective for the vasculature. Additionally, they possess natriuretic and consequent diuretic properties, achieved by direct effects on renal tubules that decrease sodium reabsorption and increase excretion [34,39]. Hyponatremia due to CCBs is rare for both dihydropyridine and non-dihydropyridine classes. Most reported cases occur with advanced age, shortly after treatment initiation, and are mainly described as isolated case reports. Some studies suggest a slightly higher risk with dihydropyridines, particularly amlodipine [34,39]. Given their widespread use and the scarcity of data linking them to hyponatremia, CCBs are generally considered safe, and in certain cases may even be recommended as an alternative antihypertensive agent in patients with hyponatremia caused by other drug classes [34,39].

#### 4.2.5. Beta-Receptor Blockers (BBs)

Data on the association between beta-blockers (BBs) and hyponatremia are scarce. A large Swedish case–control study reported that the risk of severe hyponatremia with BBs is modest (especially linked to atenolol but also bisoprolol and propranolol), and appears to be mostly confined to the initiation phase of therapy. A small persistent risk cannot be excluded for propranolol and atenolol.

The underlying mechanism remains uncertain but may involve the inhibition of renin secretion by the kidney and subsequent effects on tubular sodium reabsorption [24,34].

#### 4.2.6. Angiotensin-Converting Enzyme Inhibitors (ACEIs)

Even though evidence is mostly limited to case reports and small case series, ACEIs, most commonly enalapril, but also lisinopril, ramipril and others, have been implicated in rare cases of drug-induced hyponatremia, typically shortly after initiation [34]. Although the mechanism is not fully elucidated, ACEIs may precipitate SIADH through central pathways (central conversion of angiotensin I to angiotensin II, as described in the main mechanistic pathways section), and contribute peripherally to renal sodium loss via reduced aldosterone [15,34,35]. Angiotensin II receptor blockers (ARBs) are often selected as alternatives in cases of suspected hyponatremia induced by ACEIs [35]. Importantly, in patients with heart failure, ACEIs (often together with loop diuretics such as furosemide) constitute standard therapy and are generally associated with clinical improvement; thus, hyponatremia in this context is uncommon and should prompt evaluation for alternative or concurrent causes, rather than being assumed to result directly from ACEI use [15].

#### 4.2.7. Angiotensin Receptor Blockers (ARBs)

As previously mentioned, ARBs may represent an alternative to ACEI-induced hyponatremia by mitigating the central effects of angiotensin II and reducing the urge to drink water [66]. However, documented cases of severe hyponatremia associated with ARB use exist, most often in combination with thiazide diuretics [34]. These cases are typically identified after excluding all other potential etiologies, indicating that the drugs are the primary cause of the hyponatremic state [48]. The underlying mechanism involves the downstream pathways of the RAAS. By blocking the angiotensin II type 1 receptors (AT1), ARBs inhibit the effects of angiotensin II, consequently reducing aldosterone levels [34,48]. Additionally, the literature suggests that non-osmotic ADH release may be triggered by the reduction in blood pressure resulting from antihypertensive therapy, regardless of drug class, where baroreceptor response can override osmotic stimuli [34].

#### 4.2.8. Combination Sacubitril/Valsartan

Heart failure is a common cause of hypervolemic hyponatremia, and patients with this condition are predisposed to develop this electrolyte imbalance. Although treating the underlying heart failure is essential, sacubitril/valsartan itself has been rarely associated with drug-induced hyponatremia [67,68]. While the mechanism of valsartan has already been discussed, the hyponatremia observed in this combination is thought to be primarily related to neprilysin inhibition. Neprilysin metabolizes several vasoregulatory agents, including angiotensin II and, most importantly in this context, natriuretic peptides [68]. Consequently, neprilysin inhibitors increase the bioavailability of these agents, enhancing their sodium-losing effects in the kidneys [67,69]. In patients with pre-existing water congestion, this increased sodium loss can further elevate the risk of hyponatremia.

#### 4.2.9. Antiarrhythmic Drugs

Publications on hyponatremia induced by various classes of antiarrhythmic drugs are very limited. However, recent case reports have documented instances of hyponatremia associated with amiodarone and flecainide. Although rare, a careful assessment of the temporal sequence of events suggested that these drugs were the cause of hyponatremia [42,43].

Amiodarone exerts its effects through multiple mechanisms. It primarily blocks potassium channels but also inhibits calcium and sodium channels [43]. Although the precise mechanism linking amiodarone to hyponatremia remains unclear, it has been hypothesized that the association may involve SIADH, potentially via sensitization of kidney tissue or direct stimulation of ADH secretion through its modulation of channels in both renal and neural tissues. It has also been speculated that amiodarone may induce NSIAD through interaction with V2R [14]. Furthermore, interactions with calcium channels may exacerbate these effects [14,42,43,44]. Similar pathogenic mechanisms have been described with other antiarrhythmic drugs, such as lorcainide and propafenone [15]. Overall, amiodarone-induced hyponatremia appears to be exceptionally rare compared with other drug classes.

Concerning flecainide, it exerts its effect through blocking sodium channels in cardiac tissue [70]. Similar channels are also present in renal tubules, and it has been postulated, based on case reports, that inhibition in distal and collecting ducts could compromise sodium reabsorption [38]. Nevertheless, this mechanism remains speculative, and no direct experimental evidence confirms flecainide as a cause of hyponatremia.

### 4.3. Central Nervous System Drugs

#### 4.3.1. Antidepressants

Although not the most frequently implicated medications, antidepressants are a well-recognized and clinically significant cause of hyponatremia. Earlier meta-analyses concluded that antidepressant exposure tripled the odds of hyponatremia (OR 3.160; 95% CI 1.911–5.225), with a higher risk for SNRIs compared with SSRIs (OR 1.292; 95% CI 1.120–1.491; *p* < 0.001) and a lower risk for mirtazapine (OR 0.607; 95% CI 0.385–0.957; *p* = 0.032) [71]. A more recent systematic review and meta-analysis including 38 observational studies confirmed that both SSRIs and SNRIs significantly increase the risk of hyponatremia (SSRIs: OR 2.158; SNRIs: OR 2.270; *p* < 0.001), with SNRIs showing a slightly higher risk, particularly for clinically significant hyponatremia. Among individual drugs, fluoxetine and venlafaxine carried the greatest risk, whereas sertraline and duloxetine were linked to lower risk [72].

These findings can be complemented by real-world data from a U.S. healthcare database, which reported that, over three years, 14.3% of patients taking duloxetine and 12.4% on escitalopram developed hyponatremia, whereas the rates were 10.3% for sertraline and 8.7% for bupropion. Within the first 30 days, the overall incidence was 0.87%, increasing to 10.5% over the full three-year follow-up in the antidepressant-treated groups [73]. Differences between meta-analytic and real-world findings may reflect variations in study populations, follow-up duration, and the detection of mild versus clinically significant hyponatremia.

Consequently, cases have been documented with SSRIs and SNRIs, which are the most prevalent and lead to the highest rate of hospitalizations [74]. Additionally, tricyclic antidepressants (TCA), and other atypical compounds like mirtazapine, bupropion and trazodone are also implicated, though to a much lesser extent. This explains their use as an option for individuals predisposed to hyponatremia who require antidepressant treatment [13,15,71]. For newer antidepressants such as vortioxetine, vilazodone, and levomilnacipran, evidence regarding their association with hyponatremia remains limited [18].

Antidepressant-related hyponatremia usually emerges within the first few weeks following initiation of therapy [14,74]. This class of drugs is known to induce hypotonic hyponatremia primarily through increased water retention rather than sodium loss [33,74]. Although the precise mechanism remains unclear, generally, elevated ADH plasma levels associated with plasma hypo-osmolality and urine normo-osmolality suggest SIADH as the predominant mechanism underlying this electrolyte disturbance. Additionally, it is also associated with increased renal responsiveness to ADH and decreased threshold of osmotic regulation for ADH release [29,32,33]. These suggested mechanisms are believed to be associated with the effects of serotonin and norepinephrine on respective receptors of the hypothalamus [29,74]. However, each class of antidepressants possesses distinct characteristics that may further influence the incidence and development of hyponatremia.

SSRIs, particularly escitalopram, have been most frequently reported to cause SIADH, whereas sertraline and paroxetine are considered safer options, with fewer reported cases of this adverse effect [33,73]. Animal studies suggest serotoninergic and α-adrenergic receptors activity induced by SSRIs may play a role in stimulating ADH secretion [75]. Serotonin has also been reported to enhance renal responsiveness to ADH and to lower the osmotic threshold for ADH release [31]. SSRIs have also been associated with NSIAD through their action on V2R in the kidney [14,33]. Notably, sertraline and fluoxetine have been shown to promote renal water retention by upregulating AQP2 in rat collecting duct cells through V2R activation in the absence of ADH [14,45,76].

The incidence of hyponatremia with SNRIs is primarily associated with venlafaxine, likely due to its dual action on serotonin and noradrenaline levels. A recent study including 234,217 first-time users of an SSRI–venlafaxine combination identified a strong association with the development of profound hyponatremia, with risk increasing with age and female sex, affecting as many as 1 in 15 women aged ≥80 years [77]. The literature regarding duloxetine is contradictory and not fully understood. While some studies suggest a relatively lower risk of hyponatremia with this drug, more recent evidence has reported a much higher incidence when compared with other antidepressants [29,73,78].

Despite their lower risk of inducing hyponatremia and a mechanism that is not fully understood, tricyclic antidepressants are believed to stimulate ADH secretion via their anticholinergic action, which may also activate thirst mechanisms [28,29].

Some atypical antidepressants have also been associated with the development of this electrolyte state. Mirtazapine exerts its effects through noradrenergic and serotoninergic mechanisms. By enhancing serotonin levels and its action on 5-HT receptors, mirtazapine may induce SIADH, similarly to mechanisms observed in other antidepressant classes, although not yet fully understood [79]. However, its more specific receptor activity is believed to explain the comparatively lower risk of hyponatremia associated with mirtazapine, as opposed to other antidepressants [29,80]. Bupropion has even fewer reported cases [73]. Its mechanism is predominantly associated with dopamine and norepinephrine reuptake inhibition, with minimal effects on serotonin. The risk of bupropion alone is relatively low; however, its association with other drugs increases the risk of hyponatremia due to potential drug–drug interactions, as this drug is a strong inhibitor of CYP2D6. Since several SSRIs, SNRIs, and TCAs are metabolized via CYP2D6, co-administration with bupropion can increase the risk associated with these drugs, potentially necessitating dose reductions [74].

#### 4.3.2. Antipsychotics

Antipsychotics have also been implicated in inducing hyponatremia, although their contribution may be obscured by underlying psychiatric conditions, in which patients often exhibit compulsive water drinking, known as psychogenic polydipsia [14,15]. Antipsychotics may further exacerbate this condition through their well-known anticholinergic effects [18]. Hyponatremia has been documented to occur within two to four weeks of antipsychotic initiation or following a substantial dose increase [18].

Both atypical and typical antipsychotics have been linked to hyponatremia. An in silico pharmacodynamic analysis identified that of 139,816 reports involving at least 1 of 19 antipsychotics, 1.1% included hyponatremia as a reported adverse reaction [81]. Typical antipsychotics, such as chlorpromazine or haloperidol, are reported to have a higher risk of inducing hyponatremia compared with second-generation antipsychotics (OR 2.12; 95% CI 1.83–2.46) vs. (OR 1.3; 95% CI 1.15–1.51), and are associated with greater severity and hospitalization rates. Interestingly, although second-generation antipsychotics are more widely prescribed in the general population, the proportion of patients hospitalized for hyponatremia is similar to that observed with first-generation antipsychotics (4.0% vs. 3.8%) [14,82,83,84].

Apart from thirst-inducing mechanisms, antipsychotics have also been associated with SIADH [83]. Their D2 receptor-blocking mechanism leads to receptor supersensitivity, resulting in increased ADH release [83,85] and elevated dopamine levels, which stimulate the thirst center in the hypothalamus [86]. Animal studies regarding haloperidol have concluded that this drug also acts on inner medullary collecting duct cells as a V2R agonist [14,45,87]. Second-generation antipsychotics, like aripiprazole, clozapine, risperidone and particularly olanzapine, exhibit less affinity to D2 receptors and more potent activity at 5-HT2A, which prevents D2 receptor hypersensitivity [82,86]. This property makes these drugs more effective in preventing psychogenic polydipsia and explains their lower incidence of hyponatremia [83]. However, serotonin-mediated effects on 5-HT receptors, along with increased ADH renal responsiveness and reduced osmotic threshold for ADH regulation, as discussed earlier, might also contribute to antipsychotic-induced SIADH [31].

#### 4.3.3. Anticonvulsants

Carbamazepine (CBZ) and oxcarbazepine (OXC) are the most frequently implicated anticonvulsant drugs in hyponatremia [15]. OXC was identified as having a greater incidence (25 to 75%) than CBZ (5 to 40%) [88]. Two population-based retrospective cohort studies evaluated the 30-day risk of hospitalization for hyponatremia following prescriptions of CBZ, valproic acid, phenytoin, or topiramate, compared with nonusers. CBZ use was associated with a markedly higher 30-day risk than nonuse (0.39% vs. 0.05%; relative risk (RR) 8.20; 95% CI 5.40–12.46). Similarly, users of valproic acid, phenytoin, and topiramate also exhibited an increased risk compared with nonusers (0.17% vs. 0.06%; RR 2.62; 95% CI 1.57–4.36) [89]. Furthermore, in a cohort of 1009 OXC-treated patients, severe and symptomatic hyponatremia occurred in 11.1% and 6.8% of cases, respectively, with 2.8% being clinically significant. Additionally, hyponatremic symptoms were reported in 59.4% of patients within 2 years after starting OXC, highlighting the cumulative risk over time [90]. The increase in hyponatremia risk with treatment duration was assessed in a contemporary study [14]. The risk increased by approximately 1.3-fold with each year of OXC therapy (OR 1.33; 95% CI 1.03–1.71; *p* = 0.031) [91], and is further influenced by factors such as dose, older age, low body weight and concomitant medications [14,90,91,92].

Hyponatremia typically manifests shortly after treatment initiation, with patients often being symptomatic, although many cases were reported as asymptomatic [83,88,91,93]. Other anticonvulsants, like sodium valproate, eslicarbazepine, levetiracetam, gabapentin, or topiramate, have also been reported, although to a much lesser extent [16,27].

SIADH is considered the mechanism by which anticonvulsants lead to hyponatremia, through altered sensitivity of hypothalamic osmoreceptors [14,15,88,94]. Specifically, OXC and CBZ are also recognized for enhancing collecting duct permeability via the V2R/AQP2 pathway, independently of ADH, suggesting a drug-induced NSIAD [18,76,83]. Additionally, OXC is documented to inhibit PGE2 formation, thereby preventing its interaction with EP1 and EP3 receptors [91].

#### 4.3.4. Anxiolytics, Sedatives and Hypnotics

The literature on hyponatremia induced by these drug classes is very limited and not fully elucidated, due to many confounding factors that complicate the interpretation of evidence [18]. Nonetheless, there are some case reports on benzodiazepines and other anxiolytics, which indicate a dose-dependent risk [84,95,96]. The exact mechanism by which benzodiazepines and other hypnotic agents, such as zolpidem, induce hyponatremia remains unclear. However, these medications are known to influence the neurotransmitter GABA, which has been shown to interact with vasopressinergic neurons. This interaction supports SIADH as the underlying mechanism [84,96].

#### 4.3.5. Central Nervous System Stimulants

While most amphetamines work by inducing the vesicular release of catecholamines, such as dopamine and norepinephrine, by reversing the function of their transporters, they also exhibit serotoninergic action to a lesser extent [97]. Methylphenidate (MPH) acts mainly by blocking these transporters and indirectly increasing serotonin activity [98,99].

Although MPH and other amphetamines have not been directly associated with SIADH and the literature linking them to hyponatremia is extremely scarce, MPH has been observed to transiently elevate ADH levels [18]. This information aligns with findings on the more documented amphetamine derivative 3,4-methylenedioxymethamphetamine (MDMA) [99]. MDMA has potent serotoninergic effects and has been associated with inducing SIADH and psychogenic polydipsia, thereby exacerbating hyponatremia. MDMA does have more reports of hyponatremia induction, leading to severe and even life-threatening events [15]. This is due not only to its induction of hyperhidrosis and thirst as a consequence of hyperthermia, but also to its overstimulation of serotonin, dopamine, and norepinephrine release into the synaptic cleft while inhibiting their reuptake [26]. The excessive renal water reabsorption mediated by ADH might be compensated by physiological mechanisms like the inhibition of RAAS, leading to further sodium losses [27]. However, these efforts are typically ineffective due to elevated ADH levels, creating a vicious cycle that exacerbates hyponatremia complications if not addressed promptly [100].

#### 4.3.6. Narcotic Analgesics

Hyponatremia induced by opioid therapy is a rare adverse effect. However, in some cases, opioids have been identified as the primary cause after excluding other potential factors [18]. Although the available evidence is limited, most reports involve tramadol and codeine, which are associated with a higher risk of hospitalization due to hyponatremia shortly after treatment initiation, whereas morphine has been reported less frequently, reflecting a comparatively lower risk [101]. Among these, tramadol usage poses the highest risk, making codeine a better alternative for patients prone to developing hyponatremia [101]. One of the mechanisms involves tramadol’s inhibition of norepinephrine and serotonin reuptake, similar to SNRIs [101,102,103]. These effects on vasopressinergic neurons may stimulate ADH release [15].

Adverse effects such as hypotension or nausea, common to opioid use, are already known to induce hyponatremia. Furthermore, pain itself can stimulate ADH release [15,101]. Healthcare providers should carefully assess the benefit/risk ratio before withdrawing or switching opioids to avoid exacerbating the patient’s pain and, consequently, worsening hyponatremia [101].

#### 4.3.7. Dopaminergic Agents

The literature on hyponatremia induced by dopaminergic therapies is scarce and mainly based on case reports. In fact, hyponatremia related to dopaminergic agents is exceedingly rare, particularly considering their widespread use. Nonetheless, cases of SIADH have been reported with dopaminergic therapies, usually resolving after serum sodium correction, dose reduction, drug withdrawal, or substitution [104]. Levodopa–carbidopa, the cornerstone of Parkinson’s disease management, has been rarely associated with hyponatremia, with most cases resolving upon drug discontinuation [104].

The exact mechanism underlying dopaminergic drug-induced SIADH is not fully understood and may involve individual susceptibility. Experimental evidence suggests that D4 receptor–mediated modulation of ADH release by reducing GABAergic inhibition of supraoptic neurons may play a role [104]. Since dopamine agonists exhibit affinity for D4 receptors, they may also contribute to SIADH through this mechanism.

This mechanism aligns with reports on pramipexole, which has high affinity for D2/D3 receptors and the highest affinity for D4 receptors among dopamine agonists, potentially explaining its greater propensity to increase ADH secretion. In published cases, SIADH developed either within the first two weeks of pramipexole initiation or during dose escalation, with resolution following dose reduction or drug withdrawal [104,105]. Rotigotine has also been implicated with SIADH, occurring in a dose-dependent manner, despite its relatively lower affinity for D4 receptors [104,105,106].

Amantadine, a dopaminergic modulator, has rarely been associated with SIADH [105], with sodium levels normalizing after drug withdrawal. Although uncommon, SIADH should be recognized as a potential adverse effect of dopaminergic therapy [107].

### 4.4. Antineoplastic and Immunomodulating Agents

Electrolyte disorders induced by medication are common in cancer patients and require careful attention to prevent further complications in already debilitated patients [19,108], thereby improving overall prognosis. Hyponatremia induced by chemotherapy is frequent [30,108], but often underdiagnosed, as its symptoms can result from both supportive care medications [30] and the underlying malignancy itself [109,110].

The former can induce hyponatremia through several mechanisms, primarily SIADH. Additional contributing factors include vomiting or diarrhea, which cause both volume depletion and sodium losses (nausea might also stimulate ADH secretion), poor solute intake due to anorexia, adrenal insufficiency secondary to metastases, and other comorbidities such as infections [15,111].

Hyponatremia is more commonly observed after treatment with conventional chemotherapeutic agents, including vinca alkaloids, platinum compounds, and alkylating agents, and is less frequent with targeted therapies, although some cases have been reported [30,111]. The toxic effects of these drugs on the hypothalamus and neurohypophysis justify SIADH as the main mechanism. However, many other mechanisms are also associated, including adrenal insufficiency, hypothyroidism, renal salt wasting and increased sensitivity to ADH [30]. Continuous monitoring of electrolyte levels is essential, and clinicians should consider supplementation in patients at high risk for hyponatremia [110].

#### 4.4.1. Alkylating Agents

Hyponatremia associated with alkylating agents is primarily reported with nitrogen mustards, including ifosfamide, chlorambucil, and particularly cyclophosphamide [15,45]. SIADH is considered the primary mechanism by which alkylating agents lead to hyponatremia, although renal tubular effects have also been reported [30]. More extensive research exists on the mechanisms by which cyclophosphamide disrupts sodium homeostasis, potentially leading to severe symptoms [19]. While hyponatremia is predominantly seen at high doses, cases associated with lower doses have also been reported [15]. Cyclophosphamide-induced tumor lysis may stimulate the release of ADH or ADH-like peptides from tumor cells or the pituitary gland [19], leading to SIADH. Interestingly, some case reports of cyclophosphamide-induced hyponatremia showed normal ADH levels. This suggests that apart from central actions, cyclophosphamide and its metabolites may act at the renal level, inducing NSIAD through V2R upregulation or direct effects, as demonstrated in animal studies [14,19,30,76].

Although rare [95], cisplatin-induced hyponatremia is more frequently reported, partly because the drug is often administered with hypotonic fluids to prevent nephrotoxicity, potentially worsening prognosis [15,19]. While evidence is limited, cisplatin-induced hyponatremia through SIADH appears less common, presenting as euvolemic, whereas it occurs more frequently via renal salt wasting, resulting in hypovolemia [45,108,112]. SIADH onsets in a matter of a few days and quickly resolves after drug removal. In contrast, renal salt wasting can persist for several days after the cessation of this causative agent [30]. It is believed to involve the entire nephron since cisplatin is excreted predominantly in the urine. Cisplatin-induced damage to the proximal tubule, mainly by inhibiting the sodium-potassium pump, impairs sodium and water reabsorption. As a result, increased sodium chloride in the filtrate triggers the macula densa to decrease glomerular filtration rate to maintain nephron pressure constant, which can worsen hyponatremia. In the loop of Henle, dysfunction coupled with increased filtrate concentration disrupts the countercurrent gradient, further interfering with the loop of Henle’s physiological properties. Dysfunction at the distal convoluted tubule, likely due to DNA damage of thiazide-sensitive co-transporter genes induced by this platinum-containing compound [19], further compromises NaCl reabsorption, resulting in hyperosmotic filtrate entering the collecting duct. Even though the literature suggests that cisplatin decreases AQP2 expression, which would impair water reabsorption, volume depletion activates baroreceptors and increases ADH release in order to counter this mechanism [108].

#### 4.4.2. Vinca Alkaloids

Vinca alkaloids such as vincristine and vinblastine may induce hyponatremia within days to a few weeks after administration [19]. Vinca alkaloids present toxicity in the neurohypophysis and hypothalamus, inducing uncontrolled release of vasopressin and, consequently, representing the major cause of hyponatremia within this class [14,15,45]. Authors advise caution when using antifungal therapy concurrently with vinca alkaloids since the former inhibit the metabolism of vinca alkaloids. This inhibition leads to an increase in these chemotherapeutic agents’ levels and consequent severe neurotoxicity [15,19].

#### 4.4.3. Monoclonal Antibodies

The literature reports very few cases of monoclonal antibody-induced hyponatremia, and only a small number are associated with SIADH [30]. Anti-IGF-1R (type 1 human insulin-like growth factor receptor) monoclonal antibodies, such as cixutumumab, have been described to enhance sodium excretion [19]. Additionally, anti-VEGF (vascular endothelial growth factor) monoclonal antibodies, such as bevacizumab [113], have also been reported to induce hyponatremia because of nephrotic syndrome [19].

Immune checkpoint inhibitors have also been associated with toxicity reports [30,114]. A recent real-world pharmacovigilance analysis concluded that hyponatremia associated with PD-1/PD-L1 inhibitors (nivolumab, pembrolizumab, and atezolizumab) occurred mainly in patients aged 45 and older, with a higher incidence in males and median onset times of 42, 35 and 20 days, respectively [115]. Nivolumab, a PD-1 inhibitor, has been associated not only with SIADH but also with autoimmune hypophysitis, resulting in ACTH deficiency and SAI. In this context, cortisol deficiency impairs the physiological suppression of ADH, leading to a euvolemic hyponatremia that is clinically and analytically similar to SIADH, except for the presence of low cortisol levels, which allows the differential diagnosis. Moreover, nivolumab’s immune-mediated effects on the adrenal glands can, although less common, precipitate adrenalitis, resulting in PAI. This monoclonal antibody has also been associated with tubulointerstitial nephritis, further impairing kidney function [19,30].

Ipilimumab, representing monoclonal antibodies against cytotoxic lymphocyte protein-4 (CTLA-4) may also induce hypophysitis, cascading the same mechanisms of SAI even more commonly than PD-1 and PD-L1 inhibitors.

Additionally, central hypothyroidism following hypophysitis has also been reported for both classes of immune checkpoint inhibitors [30,55,111].

#### 4.4.4. Tyrosine Kinase Inhibitors

Although the pathophysiology remains unclear and the effects are dose dependent, the literature suggests SIADH as the main underlying mechanism. This could occur either directly influencing hypothalamic pathways, like imatinib [116], or other poorly understood mechanisms like decreasing papillary solute concentrations and increasing urine osmolality, as happens with sorafenib [30]. Gefitinib’s mechanism is not fully elucidated. However, it is believed to induce fluid retention, triggering hyponatremia. This may occur either through SIADH resulting from epidermal growth factor receptor (EGFR) pathway blockade or as a consequence of one of its most common adverse effects, such as diarrhea, which can also lead to hyponatremia, as previously discussed [109].

#### 4.4.5. Antimetabolites

Studies associating antimetabolites with hyponatremia are extremely scarce. High doses of methotrexate might induce this electrolyte disturbance due to neurotoxicity, affecting the hypothalamic region and inducing ADH secretion [15]. Activation of natriuretic peptides is another plausible mechanism [19].

### 4.5. Digestive System Drugs

#### 4.5.1. Proton Pump Inhibitors

Evidence regarding PPI-induced hyponatremia remains limited [117]. The association between proton pump inhibitors (PPIs) and hyponatremia is particularly evident with newly initiated therapy, which may lead to hospitalization. During chronic use (over one year), the risk of moderate hyponatremia increases, especially in individuals of advanced age [15,118]. Reports of hyponatremia are more frequent with omeprazole [119] and, to a lesser extent, with lansoprazole [120].

Even though the mechanism is also not fully elucidated, the assessment of hyponatremic patients with high urine osmolality and euvolemic volume status has raised suspicion of SIADH induced by PPIs for most cases [121]. However, less commonly, these drugs are also known to induce acute interstitial nephritis, leading to salt-losing nephropathy [15,117,121].

Hyponatremia occurring a long time after this therapy initiation makes it less likely to represent the cause [122]. Studies have also demonstrated that switching to a different PPI does not solve the problem. Discontinuation and considering alternative treatment should be taken into consideration to improve the patient’s condition [117,121].

#### 4.5.2. Laxatives

Cleansing bowel preparation (CBP) regimens are classified as hypertonic and isosmotic. Even though poorly tolerated due to the high volume ingested, guidelines suggest isosmotic preparations, such as polyethylene glycol, as safer for being less likely to cause fluid shifts and electrolyte imbalances [123,124,125]. Overall, even if well tolerated, various electrolyte disturbances are common around these preparations, with hyponatremia being particularly notable. In fact, it is more commonly observed with hypertonic preparations, which are also linked to higher rates of hospital admission [15,124].

Hyponatremia tends to develop through various mechanisms around CBPs. In addition to the increased water intake, SIADH induced by osmotic and non-osmotic (dehydration caused by diarrhea and nausea) stimuli stands as the main mechanism [15,126]. Additionally, pain and anxiety might be associated with these preparations and are reported to induce ADH release [15,127].

Although hyponatremia induced by CBPs is usually self-resolving, severe complications have been reported for either type of preparation [125]. Therefore, it is recommended to thoroughly investigate the medical history of patients at risk before prescribing these preparations [123].

### 4.6. Locomotor System Drugs

#### Non-Steroidal Anti-Inflammatory Drugs

Documented cases of NSAID-induced hyponatremia are particularly scarce [12,15,45]. However, these drugs can induce mild to severe hyponatremia, essentially over long periods of exposure in individuals who already have risk factors for developing this sodium disturbance [128].

The interaction between NSAIDs and prostaglandins was previously believed to be associated with SIADH [15,45]. However, recent studies have demonstrated that the potentiation of ADH effect without increasing its secretion is a more plausible mechanism, as NSAIDs remove the inhibitory effect of prostaglandins on ADH activity. Reducing PGE2 levels, in the presence of ADH, interaction with EP1 and EP3 receptors will be reduced [12,45].

### 4.7. Anti-Infective Drugs

#### 4.7.1. Sulfonamides

As previously mentioned, underlying infections can induce hyponatremia through distinct mechanisms by altering physiological conditions [15,129]. This makes it difficult for clinicians to identify hyponatremia induced by antibiotics, which is already a rare cause [15].

The combination of trimethoprim and sulfamethoxazole, also referred to as cotrimoxazole (TMP–SMX), a broad-spectrum antibiotic [130], is well-known to cause moderate to severe hyponatremia in a dose-dependent manner [131,132]. The main mechanism is renal sodium loss, which reduces effective circulating volume and triggers baroreceptor-mediated non-osmotic AVP secretion, leading to hypovolemic hyponatremia [15,131]. Renal salt wasting is primarily explained by the structural similarity of TMP–SMX to the potassium-sparing diuretic amiloride, since both block ENaC in the distal convoluted tubule and collecting duct [15,36,131,133]. Additionally, TMP–SMX induces aldosterone resistance by inhibiting aldosterone-mediated sodium reabsorption [132,133]. TMP–SMX frequently increases serum potassium levels, often causing hyperkalemia alongside hyponatremia.

Identifying the underlying mechanism is crucial to initiate an appropriate response to hyponatremia, as this condition tends to develop rapidly after onset and usually resolves within weeks after discontinuation [131,133]. It should be noted that intravenous administration of this drug often requires large daily volumes of fluid, which may exacerbate hyponatremia [130]. In cases of salt-losing nephropathy, sodium supplementation should be considered, and, for high-risk patients, alternative treatments should be investigated [36,132].

#### 4.7.2. Fluoroquinolones

Although a rare adverse effect and uncommonly documented, SIADH has been reported in a few cases regarding fluoroquinolones, more specifically, ciprofloxacin [15,57,134]. After ruling out other etiologies of euvolemic hyponatremia, it was hypothesized that the lipophilic properties of ciprofloxacin enable it to cross the blood–brain barrier, allowing ciprofloxacin to bind to glutamate receptors and stimulate ADH release. Moreover, the inhibition of GABA receptors by ciprofloxacin and other fluoroquinolones might be related to ADH release [57,134]. Additionally, other ciprofloxacin-therapy-associated adverse effects like vomiting and diarrhea might further compromise the patient’s prognosis [134].

#### 4.7.3. Oxazolidinones

There have been several cases of severe hyponatremia reported with linezolid, suggesting it is associated with SIADH, although the mechanisms by which this occurs are unclear to present research [129].

#### 4.7.4. Other Anti-Infective Drugs

A systematic review has also linked aminoglycosides and colistin to electrolyte and acid–base disturbances, including hyponatremia, with limited evidence derived from case reports. These complications are thought to stem from their nephrotoxic potential, with gentamicin posing the greatest risk. However, the mechanisms by which aminoglycosides disrupt renal tubular function remain mostly speculative, and the true prevalence of these adverse effects has not been clearly established [13,135].

The literature regarding hyponatremia induced by other anti-infective drugs, such as antifungal and antiviral therapies, is extremely limited.

Pentamidine, an antifungal agent, exerts its effects on sodium channels. On top of that, when administered rapidly, it is believed to cause a drop in blood pressure, which might activate compensatory mechanisms that increase water reabsorption [15]. Moreover, although the literature is limited, voriconazole-associated hyponatremia has been described in small case series and reports, with only a few cases reporting severe hyponatremia. It has been documented to emerge within days to a few weeks of therapy initiation and generally improves after dose reduction or discontinuation. This adverse effect appears to be primarily concentration-dependent, with a higher risk in patients exhibiting elevated trough levels. Genetic variability also plays a role, as CYP2C19 polymorphisms influence voriconazole metabolism, and poor metabolizers, who have reduced clearance, are more common in Asian populations. Consequently, Asians and older patients appear to be at greater risk. Although the mechanism is not fully understood, hyponatremia is thought to result from SIADH or salt-losing nephropathy [136,137,138,139].

The combination of nirmatrelvir–ritonavir, an antiviral, is a recently reported drug associated with hyponatremia. It has been linked to SIADH cases due to laboratory findings, volume status and symptoms initiating mere days after drug administration [20].

### 4.8. Hormones and Endocrine Diseases Drugs

#### 4.8.1. Vasopressin and Analogs

Desmopressin, a synthetic analog, has a longer half-life and stronger antidiuretic effect than endogenous ADH due to higher selectivity to V2R. Desmopressin is a particularly significant drug to induce severe hyponatremia through the same mechanisms and especially in at-risk patients [14,45,140].

Terlipressin, however, is considered safer for its higher affinity for V1R (promoting vasoconstriction effects). Despite this, it can still act on V2R and its consequent effects [141].

#### 4.8.2. Oxytocin

Oxytocin has a very similar structure to ADH [142]. For this reason and although being a hundred-fold weaker, oxytocin action on the V2R pathway is responsible for its hyponatremia cases, in particular, when administered in intravenous hypotonic fluids [15,45,142]. Reported cases of severe hyponatremia with neurological symptoms enhance the importance of monitoring electrolyte levels as well as fluid intake during this therapy [142].

#### 4.8.3. Antidiabetics

Electrolyte disturbances are common in patients with poorly controlled diabetes, as hyperglycemia can lead to hypertonic hyponatremia. However, the literature specifically addressing glucose-lowering drugs is very scarce and sometimes contradictory, though some occasional case reports have identified hypotonic hyponatremia with sulfonylureas and, to a lesser extent, some older reports with insulin, metformin and thiazolidinediones [143].

Insulin is believed to directly affect water homeostasis by inducing fluid retention. However, it has also been shown to influence sodium reabsorption and its decreased excretion through its effects on sodium channels [143]. First-generation sulfonylureas are generally more associated with hyponatremia, although not all, nor most, of the newer generations exhibit water shift disturbances. Chlorpropamide, particularly when newly initiated, has been associated with increased water reabsorption properties [143,144]. Although its effects were initially described as SIADH, studies have not found elevated ADH levels in humans and rats [45]. Nevertheless, chlorpropamide’s agonist effect on V2R was demonstrated, as well as its ability to increase the number of V2R [45,144,145].

Despite these findings, a recent study has concluded that glucose-lowering medications did not increase the risk of hospitalization associated with severe hyponatremia. In fact, it demonstrated a protective effect, after adjusting for confounding factors known to induce hyponatremia, such as diabetes itself [143]. For example, sodium-glucose co-transporter-2 (SGLT-2) inhibitors, like empagliflozin, have been suggested as potential therapeutic options given their osmotic diuretic properties and ability to enhance free-water clearance. Nevertheless, the evidence remains ambiguous, as their pharmacodynamic profile has been linked to both increased and decreased risks of hyponatremia. At present, the available data are insufficient, and no regulatory authority has approved SGLT-2 inhibitors for the treatment of SIAD [13,143,146,147].

### 4.9. Other Pharmacological Classes and Drugs

Other pharmacological classes are extremely rare to be reported as the primary cause of hyponatremia, though occasional reports have been published.

Regarding tacrolimus, although rarely seen, salt-losing nephropathy originating from the kidney grafts can lead to severe symptomatic hyponatremia. In addition to upregulation of the Na-K-2Cl co-transporter, it concurrently leads to the downregulation of mineralocorticoid receptor expression and consequent loss of aldosterone action [148,149].

Theophylline was also associated with a few reports of drug-induced hyponatremia through its inhibition of sodium reabsorption in the proximal tubule and the thick ascending limb of the loop of Henle. Moreover, SIADH was also observed in acute theophylline intoxications [15].

Although rare, hyponatremia resulting from both unfractionated and low-molecular-weight heparin has been described in the literature [150]. Both these drugs inhibit aldosterone production by reducing the number and affinity of angiotensin II receptors in the zona glomerulosa of the adrenal glands. This will result in both sodium loss and potassium retention within days after administration, with electrolyte levels normalizing quickly after discontinuation [151,152].

The literature regarding lipid-lowering agents is extremely rare and even contradictory. A recent study concluded that although statins could theoretically cause fluid retention through AQP2 upregulation (since some hyponatremia case reports were identified regarding these drugs and ezetimibe), evidence suggested an inverse association between statins and hyponatremia, indicating a possible protective effect, though data were insufficient to support such a claim [153].

Mannitol use increases serum osmolality and, consequently, induces a water shift from the cells, possibly leading to hyponatremia [15]. Despite this, unlike other hyponatremia causes, this condition results in a hypertonic state and possible brain dehydration. Healthcare providers should acknowledge that this requires different correction strategies compared with those used in hypotonic hyponatremia states [154].

#### Comparative Overview of Drug Classes

These findings emphasize that the risk of hyponatremia varies considerably across drug classes. To provide a broader comparative overview, Table 4 compares the likelihood of hyponatremia associated with different drug classes based on the reviewed literature.

## 5. Hyponatremia Management

When managing a patient with hyponatremia, treatment requires careful consideration of the benefits and risks to determine the appropriate intensity of intervention.

Hyponatremia is associated with increased morbidity and mortality rates [4]. Concerning the latter, some studies have reported inpatient mortality rates as high as 50% in patients with severe hyponatremia [155]. Life-threatening symptoms require immediate emergency treatment with 3% hypertonic saline to rapidly increase serum sodium concentration and reduce neurological sequelae from brain edema, while minimizing the risk of osmotic demyelination or fluid overload [3,15,20]. For non-emergency treatments, it is crucial for the healthcare provider to assess clinical, laboratory and medication history to accurately identify the type of hyponatremia and implement the appropriate management strategies [20].

Given the variability among sources regarding the clinical management of hyponatremia, Table 5 was adapted from the recommendations of the European Clinical Practice Guideline on the Diagnosis and Treatment of Hyponatremia [3].

Although clinical guidelines provide clear pathways for the management of hyponatremia, the literature on the management of drug-induced cases remains limited and inconsistent. Research has primarily examined major drug classes such as thiazide diuretics, psychotropics, anticonvulsants, and antineoplastic agents, while evidence for other classes is comparatively scarce.

Discontinuation of the offending agent can lead to favorable outcomes, but this approach is not always feasible or sufficient, for example, in cases of severe and symptomatic reduction in serum sodium levels, highlighting the need for active interventions and preventive strategies [14,20]. As an example, in drug-induced SIADH, discontinuation of the offending drug can be combined with fluid restriction. However, it is important to consider the elimination half-life of the agent (approximately five half-lives are required for a clinically relevant washout) [156].

To complement the guideline-based recommendations, a literature search was performed to identify therapeutic strategies specific to drug-induced hyponatremia, with emphasis on the previously described pharmacological classes. Although data remain limited for some drug categories, the main findings are summarized in Table 6, with the full details provided in the Supplementary Material (Table S1).

## 6. Conclusions

As drug-induced hyponatremia poses significant challenges in clinical practice, understanding its mechanisms, coupled with effective management strategies, can enhance patient safety.

This review provides an in-depth analysis of the pharmacological classes associated with hyponatremia, covering both the most frequently implicated and the less commonly reported agents. Additionally, it offers a clinically oriented perspective on the management of hyponatremia across different drug classes, an aspect that distinguishes it from previously published reviews and underscores its added value to the existing literature.

## Figures and Tables

**Table 1:** Comprehensive classifications of hyponatremia.

**Clinical Classification—According to symptomatology**				
Moderately severe	Nausea without vomiting; confusion; headache; cognition impairment; gait deficits; falls.			
Life threatening	Vomiting; cardiorespiratory distress; abnormal and deep somnolence; seizures; coma (Glasgow Coma Scale ≤ 8).			
**Biochemical classification—According to serum sodium concentration**				
Mild	130–135 mmol/L			
Moderate	125–129 mmol/L			
Profound	<125 mmol/L			
**Time-based classification—According to time of development**				
Acute	Development within <48 h (associated with higher risk of brain edema and neurological complications)			
Chronic	Development over ≥48 h (allowing partial cerebral adaptation to hypo-osmolality)			
**Serum osmolality-based—According to serum osmolality**				
Non-hypotonic	Hypertonic			>290 mOsm/kg
	Isotonic			275–290 mOsm/kg
Hypotonic	According to volume status	Hypovolemic	↓ H_2_O ↓↓ Na^+^	<275 mOsm/kg
		Hypervolemic	↑↑ H_2_O ↑ Na^+^	
		Euvolemic	↑ H_2_O ↔ Na^+^ excretion	
Pseudo-hyponatremia	Laboratory artifact			

↓, decreased; ↑, increased; ↓↓/↑↑, decrease/increase predominates; ↔, normal.

**Table 2:** Hypotonic hyponatremia according to volume status.

	Hypovolemic		Hypervolemic	Euvolemic
**Volume status**	↓ H_2_O ↓↓ Na^+^ (↓ effective arterial volume)		↑↑ H_2_O ↑ Na^+^ (Ascites and edema)	↑ H_2_O ↔ Na^+^ excretion
**Urine osmolality**	>100 mOsm/Kg		>100 mOsm/Kg	>100mOsm/Kg
**Urine sodium concentration**	>30 mmol/L	≤30 mmol/L	≤30 mmol/L	>30 mmol/L
**Associated diseases**	**Renal Causes** Primary adrenal insufficiency Hypoaldosteronism Kidney disease Cerebral salt wasting	**Non-renal causes** Severe diarrhea Vomiting Intense sweating Third spacing	Advanced kidney disease (urine Na^+^ > 30 mmol/L) Nephrotic Syndrome Heart Failure Cirrhosis	Low solute intake (urine osmolality < 100 mOsm/Kg and urine Na^+^ < 30 mmol/L) Excessive water intake/Primary polydipsia (urine osmolality < 100 mOsm/Kg and urine Na^+^ < 30 mmol/L) SIADH (exclusion diagnosis) * Secondary adrenal insufficiency Hypothyroidism Kidney disease Pain Nausea
**Drug-induced**	++	+		++

Abbreviations: Na^+^, Sodium; SIADH, Syndrome of Inappropriate Antidiuretic Hormone secretion. ↓, decreased; ↑, increased; ↓↓/↑↑, decrease/increase predominates; ↔, normal; +/++, prevalence indicator. * Individuals with low solute intake and excessive water consumption might present with urine osmolality < 100 mOsm/Kg and urine Na^+^ < 30 mmol/L.

**Table 3:** Drug-induced hyponatremia mechanistic overview.

Physiological System	Mechanism Overview	Mechanism Description	Drugs/Drug Classes	
**Central action**	**SIADH** **(induced ADH release)**	- Direct stimulation of pituitary - Volume depletion effects (non-osmotic release) - Pituitary stimulation by angiotensin II after conversion of angiotensin I in the brain - Decreased threshold of osmotic regulation - Serotonin and norepinephrine effects on the hypothalamus - Dopamine receptors supersensitivity - GABA influence on vasopressinergic neurons - Altered sensitivity of hypothalamic receptors - Anticholinergic effects - EGFR pathway blocking - Neurohypophysitis - Toxic effects in the hypothalamus	- **Selective Serotonin Reuptake Inhibitors** - **Serotonin and Norepinephrine Reuptake Inhibitors** - **Typical Antipsychotics** - **Atypical Antipsychotics** - **Anticonvulsants** - **MDMA (ecstasy)** - **Alkylating agents** - **Platinum-containing compounds** - **Vinca alkaloids** - **Nivolumab** - Tyrosine kinase inhibitors - Methylphenidate - Angiotensin II Receptor Blockers - Amiodarone	- Tricyclic Antidepressants - Monoamine Oxidase Inhibitors - Mirtazapine - Benzodiazepines and other hypnotics - Opioids - Dopaminergic agents - Methotrexate - Proton Pump Inhibitors - Cleansing Bowel Preparations - Ciprofloxacin - Linezolid - Pentamidine - Voriconazole - Nirmatrelvir-ritonavir - Theophylline
	**Increase in water intake**	- Thirst-inducing mechanisms - Anticholinergic effects (dry mouth)	- **Thiazides and Thiazide-like agents** - **Tricyclic Antidepressants** - **Typical Antipsychotics** - **MDMA (ecstasy)**	
	**Other hormonal pathways**	- ACTH deficiency due to autoimmune hypophysitis - Cortisol deficiency due to secondary adrenal insufficiency	- **Nivolumab** - **Ipilimumab**	
	**Non-renal sodium losses**	- Hyperhidrosis	- **MDMA (ecstasy)**	
**Renal Action**	**Co-transporter suppressed activity**	- Blocking of the Na/Cl co-transporter (NCC) - Inhibition of the Na-K-2Cl cotransporter 2 (NKCC2) - Blocking of the epithelial sodium channel (ENaC) - Inhibition of the sodium-potassium pump (Na^+^/K^+^-ATPase) - DNA damage to co-transporter genes	- **Thiazides and Thiazide-like agents** - **Cisplatin** - **Loop Diuretics** - **Potassium Sparing Diuretics** - **Calcium-channel Blockers** - **TMP–SMX (cotrimoxazole)** - Flecainide - Pentamidine - Theophylline	
	**Aldosterone suppressed activity**	- Downregulation of mineralocorticoid receptors (hypoaldosteronism) - Aldosterone antagonism	- **Potassium Sparing Diuretics** - **TMP–SMX (cotrimoxazole)** - **Tacrolimus**	
	**Induced pathologies**	- Nephrotic syndrome induction - Interstitial nephritis - Other salt-losing nephropathies	- **Bevacizumab** - **Nivolumab** - Proton Pump Inhibitors - Aminoglycosides - Colistin - Voriconazole	
	**Other mechanisms**	- Other sodium depletion mechanisms	- **Cixutumumab**	
	**NSIAD**	- Stimulating V2R, upregulating AQP2 channels in the apical membrane, independently of ADH - Increasing V2R mRNA expression - Increasing renal responsiveness to ADH	- **Selective Serotonin Reuptake inhibitors** - **Atypical Antipsychotics** - **Haloperidol** - **Oxcarbazepine/Carbamazepine** - **Cyclophosphamide** - **Vasopressin and Analogs** - **Oxytocin** - **Chlorpropamide** - Amiodarone	
	**PGE2 Pathway**	- PGT suppressed action - Inhibition of PGE2 synthesis - Reduced EP1 and EP3 receptors activation - EP4 and EP2 receptors activation	- **Thiazides and Thiazide-like agents** - **Oxcarbazepine** - **Non-steroidal Anti-inflammatory Drugs**	
	**AQP2**	- Direct upregulation of AQP2 in the apical membrane	- Cyclophosphamide	
**Peripherical action**	**RAAS**	- Direct inhibition of the Renin–Angiotensin–Aldosterone System - Inhibition of renin secretion by the kidney - Reduced number and affinity of Angiotensin II receptors - Inhibition of angiotensin-converting enzyme peripherally - Reduced aldosterone production/secretion (hypoaldosteronism) - Pituitary stimulation by angiotensin II after conversion of angiotensin I in the brain - Inhibition of neprilysin enzyme with increased natriuretic peptides bioavailability - Activation of natriuretic peptides	- **Angiotensin-converting Enzyme Inhibitors** - **Angiotensin II Receptor Blockers** - **Angiotensin Receptor Neprilysin Inhibitors** - Beta-receptor Blockers - Methotrexate - Heparin	
	**Other hormonal pathways**	- Cortisol deficiency due to primary adrenal insufficiency - Reduced cardiac output and compensatory mechanisms due to hypothyroidism	- **Nivolumab** - **Ipilimumab**	
**Other mechanisms and associated factors**	**Tumoral-induced ADH release**	- ADH or ADH-like peptides released from either tumor or pituitary gland	- **Cyclophosphamide**	
	**Transcellular cation exchange**	- Na^+^ exchange with K+ due to hypokalaemia-inducing drugs	- **Thiazides and Thiazide-like agents**	
	**Therapy concomitant fluid intake**	- Hypotonic fluids along antineoplastic treatments - Ingestion of large amounts of water due to isosmotic cleansing bowel preparations - Electrolyte shifts due to hypertonic cleansing bowel preparations - Water shifts induced by hypertonic fluid therapy	- **Cyclophosphamide** - **Cisplatin** - **Cleansing Bowel Preparations** - **Oxytocin** - **Mannitol**	
	**Drug pharmacokinetics**	- CYP induction or inhibition	- **Bupropion**	

**Drugs highlighted in bold are those most commonly identified as being implicated in hyponatremia through the indicated mechanism**. Abbreviations: ACTH, Adrenocorticotropic Hormone; ADH, Antidiuretic Hormone; AQP2, Aquaporin 2; CYP, Cytochrome P450; EGFR, Epidermal Growth Factor Receptor; EP, Prostaglandin E2 Receptor; GABA, γ-Aminobutyric acid; K^+^, Potassium; MDMA, 3,4-Methylenedioxymethamphetamine; Na^+^, Sodium; NSIAD, Nephrogenic Syndrome of Inappropriate Antidiuresis; PGE2, Prostaglandin E2; PGT, Prostaglandin Transporter; RAAS, Renin–Angiotensin–Aldosterone System; TMP–SMX, Trimethoprim-Sulfamethoxazole; V2R, V2 Receptors.

**Table 4:** Comparative likelihood of developing hyponatremia across different drug classes according to the analyzed literature.

Drug/Drug Class	Likelihood of Drug-Induced Hyponatremia	Notes
**Thiazides or Thiazide-like agents**	+++	
**Loop diuretics**	+/−	Therapeutic strategy for euvolemic and hypervolemic hyponatremia
**Potassium-sparing diuretics**	+	
**Calcium-channel blockers**	+	
**Beta-blockers**	+	
**ACE inhibitors**	+	
**Angiotensin II Receptor Blockers**	+	
**Sacubitril/valsartan**	+	
**Antiarrhythmic drugs**	+	
**Selective Serotonin Reuptake Inhibitor**	+++	
**Serotonin–Norepinephrine Reuptake Inhibitor**	+++	
**Tricyclic antidepressants**	+	
**Atypical antidepressants (mirtazapine, bupropion and trazodone)**	+	
**Antipsychotics**	++	
**Anticonvulsants**	OXC/CBZ +++ Sodium valproate, eslicarbazepine, levetiracetam, gabapentin or topiramate +	
**Anxiolytics, sedatives, hypnotics**	+	
**CNS stimulants**	+ MDMA ++	
**Narcotic analgesics**	+	Higher risk for tramadol when compared to codeine
**Dopaminergic agents**	+	
**Alkylating agents**	CYC +++ Other nitrogen mustards ++ Cisplatin ++	
**Vinca alkaloids**	++	
**Monoclonal antibodies**	++	Hyponatremia is less prevalent than conventional chemotherapeutic classes
**Tyrosine kinase inhibitors**	+	
**Antimetabolites (methotrexate)**	+	
**Proton pump inhibitors**	+	Reports of more prevalence with omeprazole
**Laxatives**	++	
**Nonsteroidal Anti-Inflammatory Drugs**	+	
**Trimethoprim–sulfamethoxazole**	+++	
**Fluoroquinolones**	+	
**Oxazolidinones (linezolid)**	++	
**Aminoglicosydes**	+	
**Colistin**	+	
**Pentamidine**	+	
**Voriconazole**	+	Higher risk for CYP2C19 polymorphism
**Nirmatrelvir–ritonavir**	+	
**Vasopressin and analogs**	Desmopressin +++ Terlipressin ++	
**Oxytocin**	++	
**Antidiabetics**	+/−	Evidence remains ambiguous for some classes
**Tacrolimus**	+	
**Theophylline**	+	
**Heparin**	+	
**Lipid lowering agents**	+/−	Possible protective effect
**Mannitol**	+	Hypertonic hyponatremia

(+, low/rare likelihood; ++, moderate likelihood; +++, high likelihood; +/−, documented cases, though a possible protective effect has also been suggested).

**Table 5:** Recommended therapeutic strategies for the hyponatremic patient according to the European Clinical Practice Guideline on diagnosis and treatment of hyponatremia [3].

Severe Symptoms		Moderately Severe Symptoms	Without Severe/Moderately Severe Symptoms	
			Acute	Chronic
- 1st hour: i.v. infusion of 150 mL 3% hypertonic saline over 20 min - Check serum Na^+^ after 20 min while repeating an infusion of 150 mL 3% hypertonic saline for the next 20 min (Repeat twice or until a target of 5 mmol/L increase in serum Na^+^)		- Assess cause (if drug-induced, see Table 6) - Single i.v. infusion of 150 mL 3% hypertonic saline or equivalent over 20 min (aim for a 5 mmol/L per 24 h increase in serum Na^+^) - If serum Na^+^ further decreases despite treating causes, follow Severe Symptoms recommendations (Guideline suggests limiting the increase in serum Na^+^ to 10 mmol/L in the first 24 h and 8 mmol/L during every 24 h thereafter, until serum Na^+^ of 130 mmol/L)	- Check possible pseudo-hyponatremia - Stop fluids, medications and other factors contributing to hyponatremia - Assess cause: - Start diagnosis-specific treatment - If the acute decrease in serum Na^+^ exceeds 10 mmol/L: single i.v. infusion of 150 mL 3% hypertonic saline or equivalent over 20 min - Check serum Na^+^ after 4 h	- Stop fluids, medications and other factors contributing to hyponatremia - Cause-specific treatment - If moderate or profound: - Avoid an increase in serum Na^+^ of >10 mmol/L during the first 24 h and >8 mmol/L during every 24 h thereafter - Check the serum Na^+^ every 6 h until the serum sodium concentration has stabilized under stable treatment (Guideline suggests against treatment with the sole aim of increasing the serum Na^+^ if mild hyponatremia)
**In case of improving symptoms after a 5 mmol/L increase in serum Na^+^ in the first hour (regardless of acute or chronic)**	**In case of NO improving symptoms after a 5 mmol/L increase in serum Na^+^ in the first hour, (regardless of acute or chronic)**			
- Stop the infusion of hypertonic saline - Infuse the smallest feasible volume of 0.9% saline until cause-specific treatment is started - Start diagnosis-specific treatment (if drug-induced, see Table 6*)* - Check serum Na^+^ after 6 and 12 h and daily afterwards until the serum Na^+^ has stabilized under stable treatment (Guideline recommends limiting the increase in serum Na^+^ to a total of 10 mmol/L during the first 24 h and an additional 8 mmol/L every 24 h thereafter until the serum sodium concentration reaches 130 mmol/L)	- Continue an i.v. infusion of 3% hypertonic saline or equivalent (aim for an additional 1 mmol/L per h increase in serum Na^+^) - Stop if: - Symptoms improve - Serum Na^+^ increases 10 mmol/L in total - Serum Na^+^ reaches 130 mmol/L - Search for cause of symptoms (if drug-induced, see Table 6); - Check serum Na^+^ every 4 h during infusion of 3% hypertonic saline or equivalent			
				**If expanded extracellular fluid**
				- Fluid restriction (Guidelines recommend against a treatment with the sole aim of increasing the serum Na^+^ in mild or moderate hyponatremia) (Guidelines recommend against vasopressin receptor antagonists and demeclocycline)
				**If SIAD**
				- Fluid restriction - Equal second line treatments: - Increase solute intake with 0.25–0.50 g/kg per day of urea - Combination of low-dose loop diuretics - Oral sodium chloride (Guidelines recommend against the use of vasopressin receptor antagonists, lithium or demeclocycline)
				**If reduced circulating volume**
				- Restore extracellular volume with i.v. infusion of 0.9% saline or a balanced crystalloid solution at 0.5–1.0 mL/kg per h. (The need for rapid fluid resuscitation overrides the risk of an overly rapid increase in serum sodium concentration)

Abbreviations: Na^+^, sodium; i.v., intravenous; SIAD, Syndrome of Inappropriate Antidiuresis.

**Table 6:** Drug-induced hyponatremia management strategies.

Class	Sub-Class/Recommendations	Clinical Presentation	Management Suggestions	References
**Antihypertensives**	**Thiazide and thiazide like agents**	**Presence of neurological symptoms**	**If euvolemic or hypervolemic, or if symptoms are severe (regardless of extracellular fluid volume)** Hypertonic saline (3% NaCl) should be immediately given intravenously. Monitor serum Na^+^ levels. Discontinue thiazide diuretic, if possible. Fluid restriction. Equal second-line treatments: ○ Increasing solute intake with 0.25–0.50 g/kg per day of urea. ○ Combination of low-dose loop diuretics and oral sodium chloride (caution should be taken with hypertensive patients). ○ High protein diet. Complete resolution can take up to 1 week. Any potassium deficiency should be corrected.	[3,14,15,47,60,61,157,158,159]
		**Absence of neurological symptoms (asymptomatic or mildly symptomatic)**	Discontinue thiazide diuretic, if possible. Monitor serum Na^+^ levels. **If euvolemic:** Fluid restriction < 1 L/day. Increase sodium intake (non-hypertensive subjects) to increase renal free water excretion. Monitor serum Na^+^ levels. **If hypovolemic:** Normal saline (0.9% NaCl solution) should be administered (or balanced crystalloid solution). Monitor serum Na^+^ levels.	[3,15,60,61,158,159,160]
		**Measures to prevent recurrent hyponatremia and correct minimal degree**	Thiazides should not be prescribed to individuals with previous history of thiazide-induced hyponatremia (especially individuals of advanced age); When thiazide use is required, such as in patients who cannot tolerate loop diuretics, lower the dose as long as hyponatremia remains minimal (129–132 mmol/L). Monitor serum Na^+^ levels within the first 2 weeks of initiating thiazide treatment in susceptible patients, particularly in high-risk groups (advanced age, female, low body weight), and at regular intervals afterwards. Prevent excess fluid intake. Do not reduce sodium intake. Take measures to prevent stress or intercurrent illnesses Avoid co-administration of medications that may cause hyponatremia, including NSAIDs, SSRIs, and anticonvulsants.	[13,60,61,159,161,162]
	**Other Anti-Hypertensives**	It has been suggested that a spironolactone dose of 25 mg is generally considered safe. Higher doses, such as 50 or 100 mg, or concurrent use with furosemide, may increase natriuresis and often lead to hyponatremia. Reducing the spironolactone dose can be a helpful clinical strategy. Switching from other antihypertensive medications to calcium channel blockers is recommended. The primary treatment for hyponatremia in patients with cardiac dysfunction involves water restriction along with angiotensin converting enzyme inhibitors and loop diuretics.		[34,157,163]
**Central Nervous System Drugs**	**General Recommendations**	**Symptomatic**	**Mild to moderate symptoms:** **If Na^+^ < 130 mmol/L:** Administration of hypertonic saline (3%), if necessary. Drug discontinuation. Vasopressin receptor antagonists (vaptans) should be considered last-line treatment. Fluid restriction (if euvolemic state). Monitor serum Na^+^ levels. **Severe symptoms:** Administration of hypertonic saline (3%). Drug discontinuation. Consider furosemide administration to prevent the kidneys from concentrating urine even in the presence of high levels of ADH. Fluid restriction (if SIADH confirmed). Monitor serum Na^+^ levels. **Na^+^ 130–135 mmol/L:** Extra salt in the diet. Reduce the dose. ○ Reassess Na^+^ levels within 1 week. ▪ If necessary, discontinuation of the drug (switch to another group of psychotropics).	[18,95]
		**Asymptomatic**	**Na^+^ 130–135 mmol/L:** Reduce the dose if psychiatrically stable. ○ Reassess Na^+^ levels within 2–4 weeks. ▪ If necessary, discontinuation of the drug. **Na^+^ 125–130 mmol/L:** Reduce the dose if psychiatrically stable. ○ Reassess Na^+^ levels within 1–2 weeks. ▪ If necessary, discontinuation of the drug. **Na^+^ < 125 mmol/L:** Consider medical admission and/or reassess Na^+^ levels within 1 week. Oral salt tablets at 6–9 g per day in 2–3 divided doses are typically used in SIADH patients. Fluid restriction (if SIADH confirmed). Consider reducing the dose or switching to an alternative medication if feasible If necessary, discontinuation of the drug.	[18,95]
		**Measures to prevent hyponatremia**	**If patient presents with risk score ≥ 2 predisposing risk factors** ([2 points]: history of hyponatremia/SIADH, diuretic use, brain injury, malnutrition, BMI < 18.5; [1 point]: female sex, age ≥ 65 years, alcohol use disorder, methamphetamine use disorder, congestive heart failure, lung cancer, treatment with SSRIs/SNRIs, carbamazepine/oxcarbazepine, or antipsychotics): Check baseline Na^+^ (or use a value from the past 3 months) before initiating a new psychiatric medication. ○ Avoid high-risk medication. ○ Use minimum effective dose, justifying benefit over risk. ○ Start medication and check Na^+^ in 2–4 weeks. Avoid prescribing psychotropic medications together with thiazide or thiazide-like diuretics. Assess and modify other medications that may contribute. Manage potentially contributing comorbidities. Educate patients about limiting overconsumption of water. Note: In certain cases, the causative medication cannot be discontinued, particularly when there is a very high risk of psychiatric destabilization.	[18,85,87]
	**Antidepressants**	**Mild cases of euvolemic hyponatremia:** Continuation of SSRI can be considered with fluid restriction + loop diuretic. If necessary, discontinue the antidepressant. **Rapidly declining sodium levels or Symptomatic hyponatremia:** Hypertonic saline (3% NaCl) Fluid restriction. Consider stopping the current antidepressant or switching to another class or agent. ○ Examples include mirtazapine, bupropion, tricyclic antidepressants, mianserin and agomelatine. Usually resolves in 2 weeks. Monitor serum Na^+^ before initiating and for 2–4 weeks after start of antidepressant in patients at risk. Note: In patients who have been receiving SSRIs or venlafaxine for an extended period, the likelihood that hyponatremia is directly attributable to these medications is low, and alternative contributing factors should be considered.		[13,29,71,74,87,95,164]
	**Antipsychotics**	Always check if psychogenic polydipsia might be the cause of hyponatremia. **Asymptomatic:** Consider fluid restriction first (first-line treatment for SIADH-related hyponatremia). **Acute and symptomatic:** Administration of 3% hypertonic saline should be performed. Consider vasopressin receptor antagonists (check for patient hepatic and renal state). The benefit/risk of withdrawing or switching the antipsychotics should be considered carefully. ○ If discontinuation is not viable, careful consideration may be given to lowering the dose or changing to an antipsychotic with a lower risk of hyponatremia, while minimizing the risk of destabilizing psychiatric symptoms. Serum Na^+^ levels should be checked periodically during treatment and following any dose adjustments.		[13,82,83,85,87,157,165,166]
	**Anticonvulsants**	Same strategies as treating SIADH, along with removing the offending medication. **Mild and Asymptomatic:** Discontinue medication or switch to another in the same category and monitor Na^+^ levels. Other measures include fluid restriction and increasing salt intake. **Symptomatic:** Rapid correction with 3% hypertonic saline. Discontinuing the medication may be considered in severe cases, particularly if they do not respond to other treatments. Changing to another medication in the same category is another option. Monitor serum Na^+^.		[88]
	**MDMA**	**Acute symptomatic hyponatremia:** 3% hypertonic saline should be administered. **SIADH:** Fluid restriction (hyponatremia induced by phenethylamines is generally caused by SIADH). **Profound hypovolemia:** 0.9% sodium chloride should be administered.		[27]
**Antineoplastics**	**General Recommendations**	**Asymptomatic patients:** Fluid restriction. If known to cause SIADH, it should be discontinued whenever possible and replaced with another agent that does not cause hyponatremia. **Severe acute hyponatremia with neurologic alterations (SIADH or hypervolemia):** Rapid infusion of 3% hypertonic saline. Fluid restriction. Discontinuation of the offending medication, if possible. **Mild to moderate hyponatremia with SIADH:** Fluid restriction. ○ If unsuccessful, pharmacological treatment with loop diuretics, urea, or vaptans should be considered. Discontinuation of the offending mediation, if possible. **Cerebral and renal salt wasting syndromes:** Volume and sodium repletion with a combined use of isotonic saline, hypertonic saline and mineralocorticoids (for example, fludrocortisone, which has potent mineralocorticoid activity that can produce significant sodium and fluid retention while increasing urinary potassium excretion).		[19,30,108,167,168]
	**Alkalating Agents**	During cyclophosphamide treatment, administration of isotonic saline solution instead of water is a suitable approach to reduce the incidence of hyponatremia. **Severely symptomatic hyponatremia (in patients with SIADH/other euvolemic states or hypervolemia)** Administration of 3% hypertonic saline (continuous infusion or bolus) Fluid restriction is especially difficult in oncology patients who need urgent cisplatin therapy, since proper hydration is essential for safe administration of the drug. Addressing hyponatremia with vasopressin receptor antagonists in cancer patients might have possible advantages, in spite of their toxicity profile. **Hypovolemic patients:** Volume and sodium chloride remain the standard of care. Fludrocortisone enhances sodium reabsorption.		[16,108,166,167]
	**Immune Checkpoint Inhibitors**	The offending agent should be discontinued whenever possible. Fluid restriction should be established in all cases of SIADH. Administration of physiological doses of glucocorticoid usually corrects hyponatremia associated with immune checkpoint inhibitor-induced hypopituitarism with involvement of both axes. Caution is necessary as rapid correction of chronic hyponatremia may lead to osmotic demyelination syndrome. In case of primary adrenal insufficiency caused by these therapies, mineralocorticoid supplementation is recommended in addition to glucocorticoids. ○ Levothyroxine should be initiated 3–5 days after starting glucocorticoid replacement to prevent an acute adrenal crisis in cases.		[30,169]
**Remaining Classes of Drugs**	Evidence is limited mostly to case reports, and no standardized management protocol has been established. Besides general clinical management, the literature mainly highlights:			
	**Cardiovascular system drugs** ○ Anti-hypertensive drugs (amlodipine, lisinopril, enalapril, losartan, amiodarone, flecanaide) Drug discontinuation or dose reduction. ○ Heart failure drugs (sacubitril/valsartan) Drug discontinuation and substitution by valsartan alone. **Central nervous system drugs** ○ Anxiolytics, sedatives and hypnotics (zolpidem) Drug discontinuation. ○ Narcotic analgesics (codeine) Drug discontinuation. The clinical risk–benefit of opioid discontinuation or substitution should be assessed, particularly since pain exacerbation may worsen hyponatremia. ○ Dopaminergic agents (levodopa–carbidopa, pramipexole, rotigotine, amantadine) Drug discontinuation, substitution or dose reduction. **Antineoplastic and immunomodulating agents** ○ Tyrosine kinase inhibitors (osimertininb, gefitinib) Drug discontinuation and substitution. **Digestive system drugs** ○ Proton pump inhibitors (esomeprazole) Drug discontinuation.		Locomotor system drugs ○ Non-steroidal anti-inflammatory drugs (meloxicam) Drug discontinuation. Anti-infective drugs ○ Sulfonamides (sulfomethoxazole) Drug discontinuation. Combining with systemic corticosteroids with mineralocorticoid effects may compensate for TMP-related hyponatremia. ○ Fluoroquinolones (ciprofloxacin) Drug discontinuation. ○ Other anti-infective drugs (nirmatrelvir–ritonavir, rifampicin, voriconazole) Drug discontinuation or dose reduction. **Hormones and endocrine disease drugs** ○ Vasopressin and analogs (vasopressin, desmopressin) Drug discontinuation or dose reduction. **Other pharmacological classes and drugs** ○ Tacrolimus Dose reduction. ○ Theophylline Drug discontinuation.	[35,36,38,39,43,48,67,69,96,101,104,106,107,109,116,121,128,131,132,134,136,137,138,140,148,170,171,172,173,174,175,176]
**General Recommendations For Clinical Community**				
Rule out all other possible or precipitating causes. ○ Overall, all potential incriminated medications should be discontinued, and their re-administration is strongly discouraged if alternatives are available. ▪ This should be sufficient in most cases of mild/non-symptomatic hyponatremia. Clinicians should implement standard monitoring protocols, with attention to serum sodium monitoring in high-risk populations. Health practitioners should be able to evaluate when medications are the primary cause of hyponatremia and appropriately manage it to ensure patient safety. Although clinical practice guidelines recommend against the use of V2 receptor antagonists, the up-to-date literature considers urea and tolvaptan the most effective second-line therapies in SIADH. Due to its bitter taste, it is advisable to dissolve urea in sweet-tasting liquids. ▪ Treatment with V2 receptor antagonists necessitates close monitoring of both renal and hepatic function. Initiating V2 receptor antagonists at a low dose is recommended in high-risk patients and monitoring of serum sodium is required. Older medications such as demeclocycline and lithium are limited by variable efficacy and/or toxicity. Special attention should be paid when the withdrawal of offending drugs and the active management of hyponatremia are combined. This therapeutic approach might lead to a rapid increase in sodium and subsequent osmotic demyelination syndrome, characterized by neurologic manifestations due to osmotic stress in brain cells. This is of particular importance in chronic hyponatremic patients, since hyponatremia’s slow development allows the brain to activate adaptive mechanisms for water loss, which mitigate brain swelling, explaining why this form is typically less symptomatic, but also predisposes patients to the referred syndrome.				[3,5,13,14,15,24,29,60,85,95,146,155,160,166,167,177,178,179,180,181,182,183]

Abbreviations: Na^+^, sodium; BMI, body mass index; SIADH, Syndrome of Inappropriate Antidiuretic Hormone secretion; SSRI, Selective Serotonin Reuptake Inhibitors; SNRI, Serotonin and Norepinephrine Reuptake Inhibitors.

## Data Availability

Not applicable.

## Supplementary Materials

The following supporting information can be downloaded at: https://www.mdpi.com/article/10.3390/jcm14186584/s1, Table S1: Drug-Induced Hyponatremia: Detailed Management Strategies.

## Abbreviations

The following abbreviations are used in this manuscript:

ACEI	Angiotensin-Converting Enzyme Inhibitor
ACTH	Adrenocorticotropic Hormone
ADH	Antidiuretic Hormone
ANP	Atrial Natriuretic Peptide
AQP2	Aquaporin 2
ARB	Angiotensin II Receptor Blocker
ARNI	Angiotensin Receptor Neprilysin Inhibitor
AT1	Angiotensin II Receptor Type 1
BB	Beta-receptor Blockers
CBP	Cleansing Bowel Preparations
CBZ	Carbamazepine
CCB	Calcium-channel Blocker
CTLA-4	Cytotoxic T Lymphocyte Associated Protein-4
CYC	Cyclophosphamide
CYP2D6	Cytochrome P450 2D6
EGFR	Epidermal Growth Factor Receptor
ENaC	Epithelial Sodium Channel
GABA	γ-Aminobutyric Acid
GFR	Glomerular Filtration Rate
IGF-1R	Type 1 Human Insulin-like Growth Factor Receptor
MDMA	3,4-Methylenedioxymethamphetamine (ecstasy)
MPH	Methylphenidate
NCC	NaCl Co-transporter
NKCC2	Na-K-2Cl Co-transporter 2
NSAID	Non-steroidal Anti-inflammatory Drug
NSIAD	Nephrogenic Syndrome of Inappropriate Antidiuresis
OXC	Oxcarbazepine
PD-1	Programmed Cell Death Protein 1
PD-L1	Programmed Death Ligand 1
PGE2	Prostaglandin E2
PGT	Prostaglandin Transporter
PPI	Proton Pump Inhibitor
RAAS	Renin–Angiotensin–Aldosterone System
SAI	Secondary Adrenal Insufficiency
SGLT-2	Sodium-glucose Co-transporter-2
SIAD	Syndrome of Inappropriate Antidiuresis
SIADH	Syndrome of Inappropriate Antidiuretic Hormone Secretion
SNRI	Serotonin and Norepinephrine Reuptake Inhibitor
SSRI	Selective Serotonin Reuptake Inhibitor
TCA	Tricyclic Antidepressant
TMP–SMX	Trimethoprim–Sulfamethoxazole (cotrimoxazole)
VEGF	Vascular Endothelial Growth Factor
