# Supplementary material for: Drug-Induced Hyponatremia: Insights into Pharmacological Mechanisms and Clinical Practice Management
Source: J Clin Med. 2025 Sep 18;14(18):6584. doi: 10.3390/jcm14186584 (PMC12471027; doi:10.3390/jcm14186584)
Supplement: Supplementary file 1 [file jcm-14-06584-s001.zip › jcm-3808497-supplementary.pdf]

Journal of
Clinical Medicine

MDPI

Review

Drug-Induced Hyponatremia: Insights Into Pharmacological Mechanisms and Clinical Practice Management

Miguel Capinha [1,†], Marta Lavrador [1,2,*,†], Joana Liberato [3], Adriana Pinheiro [3], Ana Aveiro [4], Isabel Vitória Figueiredo [1,2] and Margarida Castel-Branco [1,2]

[1] Pharmacology and Pharmaceutical Care Laboratory, Faculty of Pharmacy, University of Coimbra; Polo das Ciências da Saúde, Azinhaga de Santa Comba, 3000-548 Coimbra, Portugal; mcapinhad@gmail.com (M.C.); marta.lavrador@ff.uc.pt (M.L.); isabel.vitoria@ff.uc.pt (I.V.F.); mmcb@ci.uc.pt (M.C.-B.)

[2] Coimbra Institute for Clinical and Biomedical Research (iCBR); Polo das Ciências da Saúde, Azinhaga de Santa Comba, 3000-548 Coimbra, Portugal; marta.lavrador@ff.uc.pt (M.L.); isabel.vitoria@ff.uc.pt (I.V.F.); mmcb@ci.uc.pt (M.C.-B.)

[3] USF Serra da Lousã, Unidade Local de Saúde de Coimbra; 3200-420 Lousã, Portugal; mjliberato@ulscoimbra.min-saude.pt (J.L.); ampinheiro1@ulscoimbra.min-saude.pt (A.P.)

[4] USF As Gândras, Unidade Local de Saúde de Coimbra; 3060-318 Cantanhede, Portugal; 42263@ulscoimbra.min-saude.pt (A.A.)

* Correspondence: marta.lavrador@ff.uc.pt; Tel.: +351-239-488-400

† These authors contributed equally to this work.

Table S1. Drug-Induced Hyponatremia: Detailed Management Strategies

Class	Sub-class/ Recommendations	Clinical presentation	Management suggestions	References
ANTIHYPERTENSIVES	THIAZIDE AND THIAZIDE LIKE AGENTS	**Presence of neurological symptoms**	**<u>If euvolemic or hypervolemic or if symptoms are severe (regardless of extracellular fluid volume)</u>**<br>• **Hypertonic saline (3% NaCl)**, should be immediately given intravenously.<br>○ Continuous infusion may be considered for cases with persistent symptoms.<br>○ If necessary, intravenous furosemide (low dose) to hasten correction of hyponatremia or if volume overload is a concern.<br>• Monitor serum Na^+ levels.<br>• Discontinue thiazide diuretic, if possible.<br>• Fluid restriction.<br>• Equal second-line treatments:<br>○ Increasing solute intake with 0.25–0.50 g/kg per day of urea.<br>○ Combination of low-dose loop diuretics and oral sodium chloride (caution should be taken with hypertensive patients).<br>○ High protein diet.<br>• Complete resolution can take up to 1 week.<br>• Any potassium deficiency should be corrected.	[3,14,15,47,60,61,156–158]
		**Absence of neurological symptoms (asymptomatic or mildly symptomatic)**	• Discontinue thiazide diuretic, if possible.<br>○ Replace by calcium-chain blocker as an antihypertensive (or beta-blocker).<br>○ If a diuretic is needed, replace with a loop diuretic.<br>▪ If the loop diuretic is not tolerated, adjust the thiazide diuretic dose alone.<br>▪ If additional antihypertensive effect is needed, consider adding a potassium-sparing diuretic.<br>• Monitor serum Na^+ levels (serum Na^+ can drop within hours in vulnerable patients, and severe hyponatremia may occur within 48 hours).<br><br>**<u>If euvolemic:</u>**<br>• Fluid restriction < 1 L/day.<br>• Increase sodium intake (non-hypertensive subjects) in order to increase renal free water excretion.<br>• Monitor serum Na^+ levels.<br><br>**<u>If hypovolemic:</u>**<br>• Normal saline (0.9% NaCl solution) should be administered (or balanced crystalloid solution).<br>○ Hypertonic solutions should be avoided.<br>• Monitor serum Na^+ levels.	[3,15,60,61,157–159]

 https://doi.org/10.3390/jcm14186584

		**Measures to prevent recurrent hyponatremia and correct minimal degree**	• Thiazides should not be prescribed to individuals with previous history of thiazide-induced hyponatremia (especially the elderly population);<br>○ Switching from a thiazide to a non-diuretic antihypertensive (or to a loop diuretic, if needed has been considered the safest strategy.<br>▪ Combining it with a potassium-sparing diuretic may enhance the antihypertensive effect while posing a lower risk of hyponatremia compared with a thiazide–potassium-sparing diuretic combination.<br>• When thiazide use is required, such as in patients who cannot tolerate loop diuretics, lower the dose as long as hyponatremia remains minimal (129–132 mmol/L).<br>• Monitor serum Na^+ levels within the first 2 weeks of initiating thiazide treatment in susceptible patients, particularly in high risk groups (advanced age, female, low body weight), and at regular intervals afterwards.<br>• Prevent excess fluid intake.<br>• Don't reduce sodium intake.<br>• Take measures to prevent stress or intercurrent illnesses<br>• Avoid co-administration of medications that may cause hyponatremia, including NSAIDs, SSRIs, and anticonvulsants. .	[13,60,61,158,160,161]
	**OTHER ANTI-HYPERTENSIVES**		• It has been suggested that a spironolactone dose of 25 mg is generally considered safe. Higher doses, such as 50 or 100 mg, or concurrent use with furosemide, may increase natriuresis and often lead to hyponatremia. Reducing the spironolactone dose can be a helpful clinical strategy.<br>• Switching from other antihypertensive medications to calcium channel blockers is recommended.<br>• The primary treatment for hyponatremia in patients with cardiac dysfunction involves water restriction along with angiotensin converting enzyme inhibitors and loop diuretics.	[34,156,162]
CENTRAL NERVOUS SYSTEM DRUGS	**General Recommendations**	**Symptomatic**	**<u>Mild to moderate symptoms:</u>**<br>**If $Na^+ < 130$ mmol/L:**<br>• Administration of **hypertonic saline (3%)**, if necessary.<br>• Drug discontinuation.<br>• Vasopressin receptor antagonists (vaptans) should be considered last-line treatment.<br>• Fluid restriction (if euvolemic state).<br>• Monitor serum Na^+ levels.<br><br>**<u>Severe symptoms:</u>**<br>• Administration of **hypertonic saline (3%)**.<br>• Drug discontinuation.<br>• Consider furosemide administration, to prevent the kidney from concentrating urine even in the presence of high levels of ADH.<br>• Fluid restriction (if SIADH confirmed).<br>• Monitor serum Na^+ levels.<br><br>**<u>Na^+ 130 – 135 mmol/L:</u>**<br>• Extra-salt in the diet.<br>• Reduce the dose.<br>○ Reassess Na^+ levels within 1 week.<br>▪ If necessary, discontinuation of the drug (switch to another group of psychotropics).	[18,95]

 https://doi.org/10.3390/jcm14186584

	**Asymptomatic**	<u>**Na^+ 130 – 135 mmol/L:**</u><br>• Reduce the dose if psychiatrically stable.<br>○ Reassess Na^+ levels within 2 – 4 weeks.<br>▪ If necessary, discontinuation of the drug.<br><br><u>**Na^+ 125 – 130 mmol/L:**</u><br>• Reduce the dose if psychiatrically stable.<br>○ Reassess Na^+ levels within 1-2 weeks.<br>▪ If necessary, discontinuation of the drug.<br><br><u>**Na^+ < 125 mmol/L:**</u><br>• Consider medical admission and/or reassess Na^+ levels within 1 week.<br>• Oral salt tablets at 6–9 g per day in 2-3 divided doses is typically used in SIADH patients.<br>• Fluid restriction (if SIADH confirmed).<br>• Consider reducing the dose or switching to an alternative medication if feasible<br>• If necessary, discontinuation of the drug.	[18,95]
	**Measures to prevent hyponatremia**	<u>**If patient presents with risk score ≥2 predisposing risk factors ([2 points]: history of hyponatremia/SIADH, diuretic use, brain injury, malnutrition, BMI <18.5; [1 point]: female sex, age ≥65 years, alcohol use disorder, methamphetamine use disorder, congestive heart failure, lung cancer, treatment with SSRIs/SNRIs, carbamazepine/oxcarbazepine, or antipsychotics:**</u><br>• Check baseline Na^+ (or use a value from the past 3 months) before initiating a new psychiatric medication.<br>○ Avoid high risk medication.<br>○ Use minimum effective dose justifying benefit over risk.<br>○ Start medication and check Na^+ in 2 – 4 weeks.<br><br>• Avoid prescribing psychotropic medications together with thiazide or thiazide-like diuretics (well-described risk factor for severe hyponatremia).<br>• Assess and modify other medications that may contribute.<br>○ Substitution with medication that is less likely to cause SIADH should be considered.<br>• Manage potentially contributing comorbidities.<br>• Educate patients about limiting overconsumption of water.<br><br>*Note: In certain cases, the causative medication cannot be discontinued, particularly when there is a very high risk of psychiatric destabilization.*	[18,85,87]
ANTIDEPRESSANTS	<u>**Mild cases of euvolemic hyponatremia:**</u><br>• Continuation of SSRI can be considered with fluid restriction + loop diuretic.<br>• If necessary, discontinue the antidepressant.<br><br><u>**Rapidly declining sodium levels or Symptomatic hyponatremia:**</u><br>• **Hypertonic saline (3% NaCl)**<br>• Fluid restriction.<br>• Consider stopping the current antidepressant or switch to another class or agent.<br>○ Examples include mirtazapine, bupropion, tricyclic antidepressants, mianserin and agomelatine.		[13,29,71,74,87,95,163]

https://doi.org/10.3390/jcm14186584

	▪ Mirtazapine, bupropion or tricyclic antidepressants may be considered as alternatives in patients at higher risk like patients taking SSRI and SNRI.<br>▪ Mianserin has been suggested in recent case reports as an alternative.<br>▪ Agomelatine has been suggested as a safe and effective treatment alternative for patients of advanced age with major depressive disorder who previously experienced hyponatremia while taking SSRIs.<br>◦ Usually resolves in 2 weeks.<br>• Monitor serum Na^+ before initiating and for 2-4 weeks after start of antidepressant in patients at risk (for all antidepressants but mainly SSRI and SNRI).<br><br>*Note: In patients who have been receiving SSRIs or venlafaxine for an extended period, the likelihood that hyponatremia is directly attributable to these medications is low, and alternative contributing factors should be considered*	
**ANTIPSYCHOTICS**	• Always check if psychogenic polydipsia might be the cause of hyponatremia.<br>◦ If so, restrict fluids and evaluate the therapeutic regimen.<br>◦ Avoid or reduce drugs known to induce dry mouth.<br><br>**Asymptomatic:**<br>• Consider fluid restriction firstly (first-line treatment for SIADH-related hyponatremia).<br><br>**Acute and symptomatic:**<br>• Administration of **3% hypertonic saline** should be performed.<br>• Consider vasopressin receptor antagonists (check for patient hepatic and renal state).<br>• The benefit/risk of withdrawing or switching the antipsychotics should be considered carefully.<br>◦ If discontinuation is not viable, careful consideration may be given to lowering the dose or changing to an antipsychotic with a lower risk of hyponatremia, while minimizing the risk of destabilizing psychiatric symptoms.<br>▪ Second-generation prescription should be prioritized, like aripiprazole (less likely to induce hyponatremia).<br>▪ Lithium has been reported to provide some benefit by helping to stabilize both sodium levels and psychiatric symptoms.<br>• Serum Na^+ levels should be checked periodically during treatment and following any dose adjustments.	[13,82,83,85,87,156,164,165]
**ANTICONVULSANTS**	• Same strategies as treating SIADH along with removing the offending medication.<br><br>**Mild and Asymptomatic:**<br>• Discontinue medication or switch to another in the same category and monitor Na^+ levels.<br>• Other measures include fluid restriction and increasing salt intake.<br><br>**Symptomatic:**<br>• Rapid correction with **3% hypertonic saline**.<br>• Discontinuing the medication may be considered in severe cases, particularly if they do not respond to other treatments.<br>• Changing to another medication in the same category is another option.<br>◦ If the patient's seizures are controlled by an anticonvulsant which is the cause of hyponatremia and changing is not feasible, management of hyponatremia along with continuation of the medication might be necessary.<br>• Monitor serum Na^+.	[88]

 https://doi.org/10.3390/jcm14186584

	MDMA	**<u>Acute symptomatic hyponatremia:</u>**<br>• **3% hypertonic saline** should be administered.<br><br>**<u>SIADH:</u>**<br>• Fluid restriction (hyponatremia induced by phenethylamines is generally caused by SIADH).<br><br>**<u>Profound hypovolemia:</u>**<br>• 0.9 % sodium chloride should be administered.	[27]
ANTINEOPLASTICS	General Recommendations	**<u>Asymptomatic patients:</u>**<br>• Fluid restriction.<br>• If known to cause SIADH, it should be discontinued whenever possible and replaced with another agent that does not cause hyponatremia.<br><br>**<u>Severe acute hyponatremia with neurologic alterations (SIADH or hypervolemia):</u>**<br>• Rapid infusion of **3% hypertonic saline**.<br>• Fluid restriction.<br>• Discontinuation of the offending medication, if possible.<br><br>**<u>Mild to moderate hyponatremia with SIADH:</u>**<br>• Fluid restriction.<br>○ If unsuccessful, pharmacological treatment with loop diuretics, urea, or vaptans should be considered.<br>• Discontinuation of the offending mediation, if possible.<br><br>**<u>Cerebral and renal salt wasting syndromes:</u>**<br>• Volume and sodium repletion with a combined use of isotonic saline, hypertonic saline and mineralocorticoids (for example, fludrocortisone, which has potent mineralocorticoid activity that can produce significant sodium and fluid retention while increase urinary potassium excretion).	[19,30,108,166,167]
	ALKALATING AGENTS	• During cyclophosphamide treatment, administration of isotonic saline solution instead of using water is a suitable approach to reduce the incidence of hyponatremia.<br><br>**<u>Severely symptomatic hyponatremia (in patients with SIADH/other euvolemic states or hypervolemia)</u>**<br>• Administration of 3% hypertonic saline (continuous infusion or bolus)<br>• Fluid restriction is especially difficult in oncology patients who need urgent cisplatin therapy, since proper hydration is essential for safe administration of the drug:<br>○ Correct hyponatremia as rapidly as possible and then continue with cisplatin therapy.<br>○ Another approach is to prioritize cancer treatment first, as this may help improve sodium levels, while closely monitoring for hyponatremia if the cancer therapy is considered more urgent.<br>• Addressing hyponatremia with vasopressin receptor antagonists in cancer patients might have possible advantages, in spite of their toxicity profile.<br>○ Patients receiving platinum-based chemotherapy can proceed without added risk of worsening hyponatremia.<br>○ For those not undergoing chemotherapy, these medications may help lower the likelihood of hyponatremia and alleviate its symptoms.<br><br>**<u>Hypovolemic patients:</u>**<br>• Volume and sodium chloride remain the standard of care.	[16,108,165,166]

	• Fludrocortisone enhances sodium reabsorption.	
IMMUNE CHECKPOINT INHIBITORS	• The offending agent should be discontinued whenever possible.<br>• Fluid restriction should be established in all cases of SIADH.<br>• Administration of physiological doses of glucocorticoid usually corrects hyponatremia associated with immune checkpoint inhibitor-induced hypopituitarism with involvement of both axes. Caution is necessary as rapid correction of chronic hyponatremia may lead to osmotic demyelination syndrome.<br>▪ Gradually increasing glucocorticoid doses has been suggested by some authors to prevent this risk of osmotic demyelination syndrome in patients with hyponatremia caused by central adrenal insufficiency.<br>○ In case of primary adrenal insufficiency caused by these therapies, mineralocorticoid supplementation is recommended in addition to glucocorticoids.<br>○ Levothyroxine should be initiated 3–5 days after starting glucocorticoid replacement to prevent an acute adrenal crisis in cases.	[30,168]
REMAINING CLASSES OF DRUGS	Evidence is limited mostly to case reports, and no standardized management protocol has been established. Besides general clinical management, literature mainly highlights:<br>• <u>Cardiovascular system drugs</u><br>○ **Anti-hypertensive Drugs (amlodipine, lisinopril, enalapril, losartan, amiodarone, flecanaide)**<br>Drug discontinuation or dose reduction.<br>○ **Heart failure drugs (sacubitril/valsartan)**<br>Drug discontinuation and substitution by valsartan alone.<br>• <u>Central nervous system drugs</u><br>○ **Anxiolytics, sedatives and hypnotics (zolpidem)**<br>Drug discontinuation.<br>○ **Narcotic analgesics (codeine)**<br>Drug discontinuation.<br>The clinical risk–benefit of opioid discontinuation or substitution should be assessed, particularly since pain exacerbation may worsen hyponatremia.<br>○ **Dopaminergic agents (levodopa-carbidopa, pramipexole, rotigotine, amantadine)**<br>Drug discontinuation, substitution or dose reduction.<br>• <u>Antineoplastic and immunomodulating agents</u><br>○ **Tyrosine kinase inhibitors (osimertininb, gefitinib)**<br>Drug discontinuation and substitution.<br>• <u>Digestive system drugs</u><br>○ **Proton pump inhibitors (esomeprazole)**<br>Drug discontinuation.<br>• <u>Locomotor system drugs</u><br>○ **Non-steroidal anti-inflammatory drugs (meloxicam)**<br>Drug discontinuation.<br>• <u>Anti-infective drugs</u><br>○ **Sulfonamides (sulfomethoxazole)**<br>Drug discontinuation.<br>Combining with systemic corticosteroids with mineralocorticoid effects may compensate for TMP-related hyponatremia.<br>○ **Fluoroquinolones (ciprofloxacin)**<br>Drug discontinuation.<br>○ **Other anti-infective drugs (nirmatrelvir-ritonavir, rifampicin, voriconazole)**<br>Drug discontinuation or dose reduction.<br>• <u>Hormones and endocrine diseases drugs</u><br>○ **Vasopressin and analogs (vasopressin, desmopressin)**<br>Drug discontinuation or dose reduction.<br>• <u>Other pharmacological classes and drugs</u><br>○ **Tacrolimus**<br>Dose reduction.<br>○ **Theophylline**<br>Drug discontinuation.	[35,36,38,39,43,48,67,69,96,101,104,106,107,109,116,120,128,131,132,134,136–138,140,148,169–175]

 https://doi.org/10.3390/jcm14186584

GENERAL RECOMMENDATIONS FOR CLINICAL COMMUNITY

- Rule out all other possible or precipitating causes.
    - Overall, all potential incriminated medications should be discontinued, and their re-administration is strongly discouraged, if alternatives are available.
        - This should be sufficient in most cases of mild/non-symptomatic hyponatremia.

- Clinicians should implement standard monitoring protocols, with particular attention to serum sodium monitoring in high-risk populations.

- Health practitioners should be able to evaluate when medications are the primary cause of hyponatremia and appropriately managing it to ensure patient safety by:
    - Drug dosing adjustment.
    - Analyzing possible interactions.
    - Advising safer alternatives.
    - Weighing the benefits associated with discontinuation against the risks of the lack of such medication.
    - Exercising with caution when prescribing medications (specially in patients of advanced age).
        - Inappropriate prescribing can be detected using explicit or implicit prescribing indicators. An example is the Screening Tool of Older People's Prescriptions (STOPP) criteria where it is recommended not to use thiazide diuretics or selective serotonin reuptake inhibitors in patients at risk.
    - Educating patients in water ingestion, symptomatology recognition and regular monitoring of serum sodium concentration.

- Although clinical practice guidelines recommend against the use of V2 receptor antagonists, up-to-date literature considers urea and tolvaptan the most effective second-line therapies in SIADH.

Recent studies describe oral urea as a safe, effective, and affordable option for SIAD, despite the lack of randomized trials. However, due to its bitter taste, it is advisable to dissolve urea in sweet-tasting liquids.

- Vasopressin receptor antagonists induce a solute-free water diuresis by inhibiting the ADH activity in the renal collecting duct. Usually initiated at lower doses, these agents have been reported to be effective in normalizing serum sodium levels in patients with SIADH, with tolvaptan generally demonstrating a favorable safety profile. However, caution is required as the risk of overly rapid sodium correction (even at a low dose) has been identified, especially in severe hyponatremia. Additional concerns include reports of hepatotoxicity and renal toxicity, notably with higher doses.
    - Treatment with V2 receptor antagonists necessitates close monitoring of both renal and hepatic function.
    - Initiating V2 receptor antagonists with low dose is recommended in high-risk patients and monitoring of serum sodium is required.

[3,5,13–15,24,29,60,85,95,146,155,159,165,166,176–182]

- Older medications such as demeclocycline and lithium are limited by variable efficacy and/or toxicity.
    - Demeclocycline has delayed onset and carries risks of nephrotoxicity.
    - Lithium is dangerous in overdose (requires regular monitoring).

- **Special attention should be paid when the withdrawal of offending drugs and the active management of hyponatremia are combined. This therapeutic approach might lead to rapid increase of sodium and subsequent osmotic demyelination syndrome, characterized by neurologic manifestations due to osmotic stress in brain cells. This is of particular importance in chronic hyponatremic patients, since hyponatremia's slow development allows the brain to activate adaptative mechanism for water loss which mitigate brain swelling, explaining why this form is typically less symptomatic, but also predisposing patients to the referred syndrome.**
    - The use of desmopressin has been proposed to reverse or prevent overcorrection.

Abbreviations: Na^+, sodium; BMI, body mass index; SIADH, Syndrome of Inappropriate Antidiuretic Hormone secretion; SSRI, Selective Serotonin Reuptake Inhibitors; SNRI, Serotonin and Norepinephrine Reuptake Inhibitors

 https://doi.org/10.3390/jcm14186584
